# Consequences of insecticide overuse in Hungary: assessment of pyrethroid resistance in *Culex pipiens* and *Aedes albopictus* mosquitoes

**DOI:** 10.1186/s13071-024-06635-5

**Published:** 2025-01-16

**Authors:** Rebeka Csiba, Zsaklin Varga, Dorina Pásztor, Bianka Süle, Vera Ihuoma Ogoke Mxinwa, Zoltán Soltész, Brigitta Zana, Krisztián Bányai, Gábor Kemenesi, Kornélia Kurucz

**Affiliations:** 1 National Laboratory of Virology, Szentágothai Research Centre, University of Pécs, Pécs, Hungary; 2 Institute of Biology, Faculty of Sciences, University of Pécs, Pécs, Hungary; 3 National Laboratory for Health Security, HUN-REN Centre for Ecological Research, Budapest, Hungary; 4 Institute of Ecology and Botany, HUN-REN Centre for Ecological Research, Vácrátót, Hungary; 5 Department of Pharmacology and Toxicology, University of Veterinary Medicine, Budapest, Hungary

**Keywords:** Asian tiger mosquito, Common house mosquito, AIM, IVM, Deltamethrin, L1014F, V1016G, F1534C, Genotyping

## Abstract

**Background:**

Mosquitoes, as vectors of various pathogens, have been a public health risk for centuries. Human activities such as international travel and trade, along with climate change, have facilitated the spread of invasive mosquitoes and novel pathogens across Europe, increasing the risk of mosquito-borne disease introduction and their spread. Despite this threat, mosquito control in Hungary still relies predominantly on chemical treatments, which poses the risk of developing insecticide resistance in local populations. While pyrethroid resistance has been documented in several countries, there is no information on this issue from Hungary. This study aims to investigate the presence of resistance in Hungarian mosquito populations by analyzing a native, already known disease vector and a recently established invasive species with public health significance.

**Methods:**

We assessed the presence of knockdown resistance (*kdr*) mutations L1014F in *Culex pipiens* and V1016G and F1534C in *Aedes albopictus* mosquitoes, which are responsible for pyrethroid resistance. Mosquito specimens were investigated retrospectively, collected from previous years within the framework of local monitoring programs run in urban areas representing five regions of Hungary. The mutations in mosquitoes were detected individually by allele-specific polymerase chain reaction (PCR) and gel electrophoresis, following generally used protocols.

**Results:**

In *Cx. pipiens*, the *kdr* mutation was detected across all five collection sites, with resistance allele frequencies ranging from 18.1% to 36.3%. Resistance alleles were identified in homozygosity and heterozygosity with the susceptible allele, resulting in 53% of the investigated mosquitoes showing resistance to pyrethroids in the Hungarian populations. In contrast, for *Ae. albopictus*, the analyzed individuals were found to carry only the susceptible alleles, indicating a homozygous susceptible genotype across the investigated populations on the basis of V1016G and F1534C genes.

**Conclusions:**

Our work highlights the consequences of the unilateral and long-term use of chemical treatments on mosquitoes. This indicates an urgent need for a change of concept in mosquito control strategy in Hungary, as well as in countries where mosquito control still relies dominantly on insecticides. The restricted use of chemical treatment is highly recommended to prevent the development of pyrethroid resistance in recently established populations of the invasive *Ae. albopictus*, and to decrease the public health risk of vector-borne diseases.

**Graphical Abstract:**

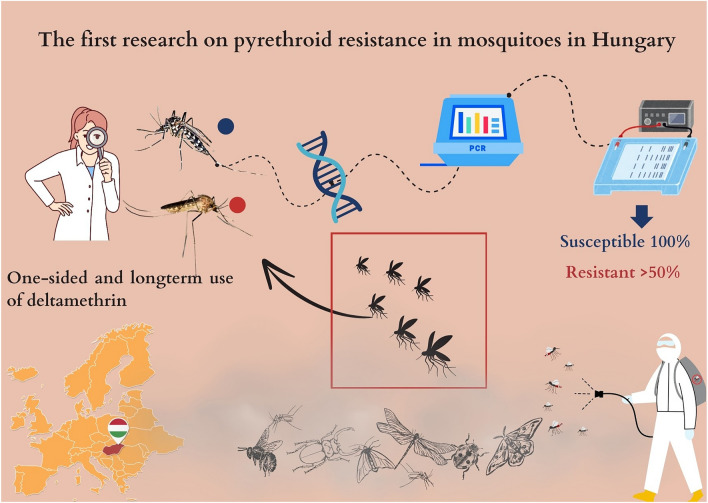

**Supplementary Information:**

The online version contains supplementary material available at 10.1186/s13071-024-06635-5.

## Background

For centuries, mosquitoes as vectors of various pathogens have presented a significant global health challenge, promoting numerous efforts to control their populations. In our increasingly globalized world, human activities such as international travel and trade facilitate the nearly limitless spread of nonnative invasive mosquito species and novel pathogens across continents and countries. Climate change further facilitates the establishment of invasive mosquitoes and enables pathogens to survive in new territories, ultimately increasing the risk of mosquito-borne diseases [1]. A holistic research approach to investigate the problem is essential to mitigate the related public health risk and apply appropriate techniques to control vector populations [2]. As a leading example of such an effort, the concept of integrated vector management (IVM) considers surveillance data, emphasizes communication between stakeholders, and encompasses a variety of treatment methods for different purposes, including physical, biological, and chemical strategies [3, 4]. While physical control focuses on eliminating or modifying artificial breeding sites of mosquitoes and incorporates personal protection measures to prevent mosquito bites, biological control aims to reduce the number of mosquito larvae and prevent their development into adults. This treatment utilizes a larvicide toxin produced by *Bacillus thuringiensis israelensis* (Bti), a naturally occurring soil bacterium. Both physical and biological methods are highly effective in reducing mosquito populations at their source. In contrast, the chemical method aims to eliminate adult mosquitoes.

Although chemical control has a long history and has been widely used to control mosquito populations, it has several technical and biological drawbacks. In the European Union, pyrethroids have been the only licensed substances for adult insect control since 2010 [5]. Besides the registered use, it is important to highlight that, as a neurotoxin, pyrethroids have a nonselective effect, meaning they can also reduce populations of other nontarget insects, show high toxicity to aquatic life, and potentially harm vertebrates [6–8]. Hence, limited use of chemical treatment and extreme caution are required when using them, particularly around water surfaces [8, 9]. In 2020, aerial spraying of pyrethroids was banned across the EU owing to its imprecise application from the air and the associated risk to nearby water bodies. Also, the commonly used fogging from trucks (ground application of chemical control) should be conducted after sunset to minimize harm to diurnal insects and because the active substance is photosensitive [10]. This technology is limited to urban areas but is inefficient in densely built-up environments, which may require more frequent treatments. However, it is crucial to avoid overuse or unilateral application of any control methods. The excessive or one-sided use of chemical substances can exert strong selection pressure on mosquito populations, leading to the development of resistance that can manifest as changes in behavior, epidermal structure, metabolic enzyme activity, and genetic mutations [11–13]. In resistant populations, chemical control becomes less effective or may fail to impact the targeted mosquito population at all. According to the recommendations of the European Commission and the European Center for Disease Prevention and Control (ECDC), chemical control should be employed only in targeted and necessary cases, such as during an epidemic caused by mosquito-borne diseases, to control vector populations [14–17].

Most mosquito-borne diseases are associated with *Culex* and invasive *Aedes* mosquito species. For instance, *Culex pipiens* (Linnaeus, 1758), a species native to Europe and commonly found in urban environments, can transmit several mosquito-borne pathogens. It is the primary vector for West Nile virus (WNV), Usutu virus, Sindbis virus, and Rift Valley fever virus [18–21]. Notably, WNV has caused numerous outbreaks in European countries, including Hungary [22–24]. Hungary is an important ecological niche for the WNV lineage 2, since its first European detection can be linked to the country in 2004. Furthermore, a comprehensive study proved that the virus strains circulating in Central and southeastern Europe continued to spread from Hungary after the first detection. Also, *Aedes albopictus* (Skuse, 1894*)* (Asian tiger mosquito), the most widespread invasive species in Europe, serves as a vector of several exotic pathogens, including dengue virus, chikungunya virus, and Zika virus [25, 26]. The rapid increase in autochthonous cases of dengue infections in Europe is associated with the presence of stable populations of the tiger mosquito in the continent [27–29]. The public health concern is also relevant in Hungary, where *Cx. pipiens* is distributed countrywide, and *Ae. albopictus* has spread to several regions of the country since its first emergence in 2014 [30]. However, the IVM approach is still not established, and mosquito control in Hungary currently relies mostly on chemical intervention [31]. Not only does this situation require an urgent change in the control approach in Hungary but, looking ahead, it is also crucial to prevent the development of insecticide resistance, as this would severely compromise the ability to control vector populations during epidemics.

To date, there is no information on pyrethroid resistance in mosquitoes circulating in Hungary, although one of the most significant genetically diverse hotspot of West Nile virus is this region. Therefore, this study aims to investigate knockdown resistance (*kdr*) mutations in *Cx. pipiens,* one of the most common native species in Hungary and a primary target of the commonly used chemical treatments, and in *Ae. albopictus*, a widespread and recently established invasive species in the country. In this study, we intend to highlight the importance of monitoring the resistance in vector populations with public health importance and fill the data gap from our region.

## Methods

### Study sites and mosquito samples

**Fig. 1:**
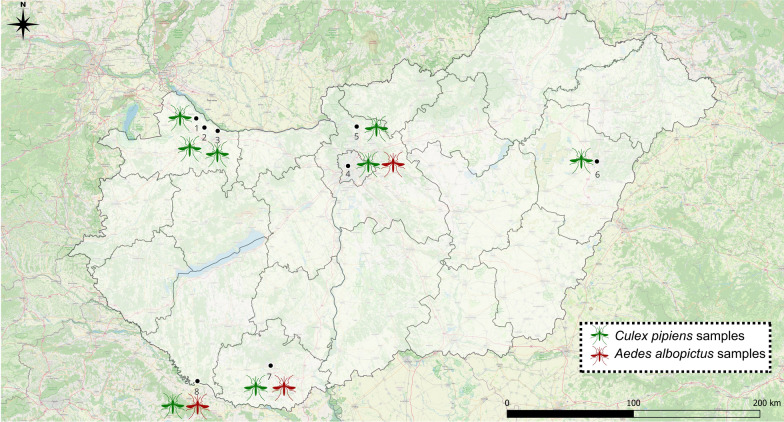
Sampling sites of investigated mosquitoes (*Culex pipiens* and *Aedes albopictus* specimens) in Hungary, 1–3: Győr-Moson-Sopron County, 4–5: Pest County, 6: Hajdú-Bihar County, 7: Baranya County, 8: Somogy County. The map was created using the QGIS free software version 3.34.13 (https://www.qgis.org)

Mosquito samples were investigated retrospectively; the specimens were collected from urban environments, in the frame of local mosquito monitoring programs during 2021–2023 (captured with CO_2_-baited Heavy-Duty Encephalitis Vector Survey (EVS) traps (Bioquip, Rancho Dominguez, CA, USA), and BG-Sentinel traps (Biogents, Germany)), identified by morphological characteristics at the species level [32–34] and stored at −20 °C until further laboratory processes. Altogether, *n* = 302 *Cx. pipiens* and *n* = 120 *Ae. albopictus* specimens were selected for our study, representative of five different counties of Hungary: Győr-Moson-Sopron, Pest, Hajdú-Bihar, Baranya, and Somogy county (Fig. 1, Table 1; for detailed sampling information on investigated specimens, see Additional file 1: Dataset S1.). As a retrospective investigation, owing to the different amounts of samples available and suitable for our study, it was impossible to analyze each region or mosquito species in equal proportions. Since the national centralized mosquito control strategy has been applied in all the investigated regions for several years, we evaluated our results at the species level.

**Table 1:** Number of *Culex pipiens* and *Aedes albopictus* mosquito specimens involved in the analysis, originating from different counties of Hungary and collected in different years

County	Sampling site number on the map (Fig. 1)	No. of investigated mosquito individuals				Total (*n*)
		(Sampling year/mosquito species)				
			2021	2022	2023	
Győr-Moson-Sopron	(1, 2, 3)	*Cx. pipiens*			100	100
Pest	(4, 5)	*Cx. pipiens*		20	6	26
		*Ae. albopictus*		20		20
Hajdú-Bihar	(6)	*Cx. pipiens*		5		5
Baranya	(7)	*Cx. pipiens*		24	100	124
		*Ae. albopictus*		4	46	50
Somogy	(8)	*Cx. pipiens*	42	5		47
		*Ae. albopictus*	31	19		50
Total (n)		*Cx. pipiens*	42	54	206	302
		*Ae. albopictus*	31	43	46	120

### Genomic DNA extraction

Specimens were processed individually (one by one separately). For genomic DNA extraction, the entire body of the mosquitoes was homogenized manually in 200 µL sterile water, using sterile quartz sand and sterile plastic tissue disruptor sticks. Nucleic acid was extracted with Quick-DNA^™^ Miniprep Plus Kit (Zymo Research, Irvine, USA), and with QIAamp^®^ Viral RNA Mini Kit (QIAGEN, Hilden, Germany), according to the manufacturers’ protocols (see Additional file 1: Dataset S1.).

### Detection of *kdr* mutation L1014F in *Culex pipiens*

**Fig. 2:**
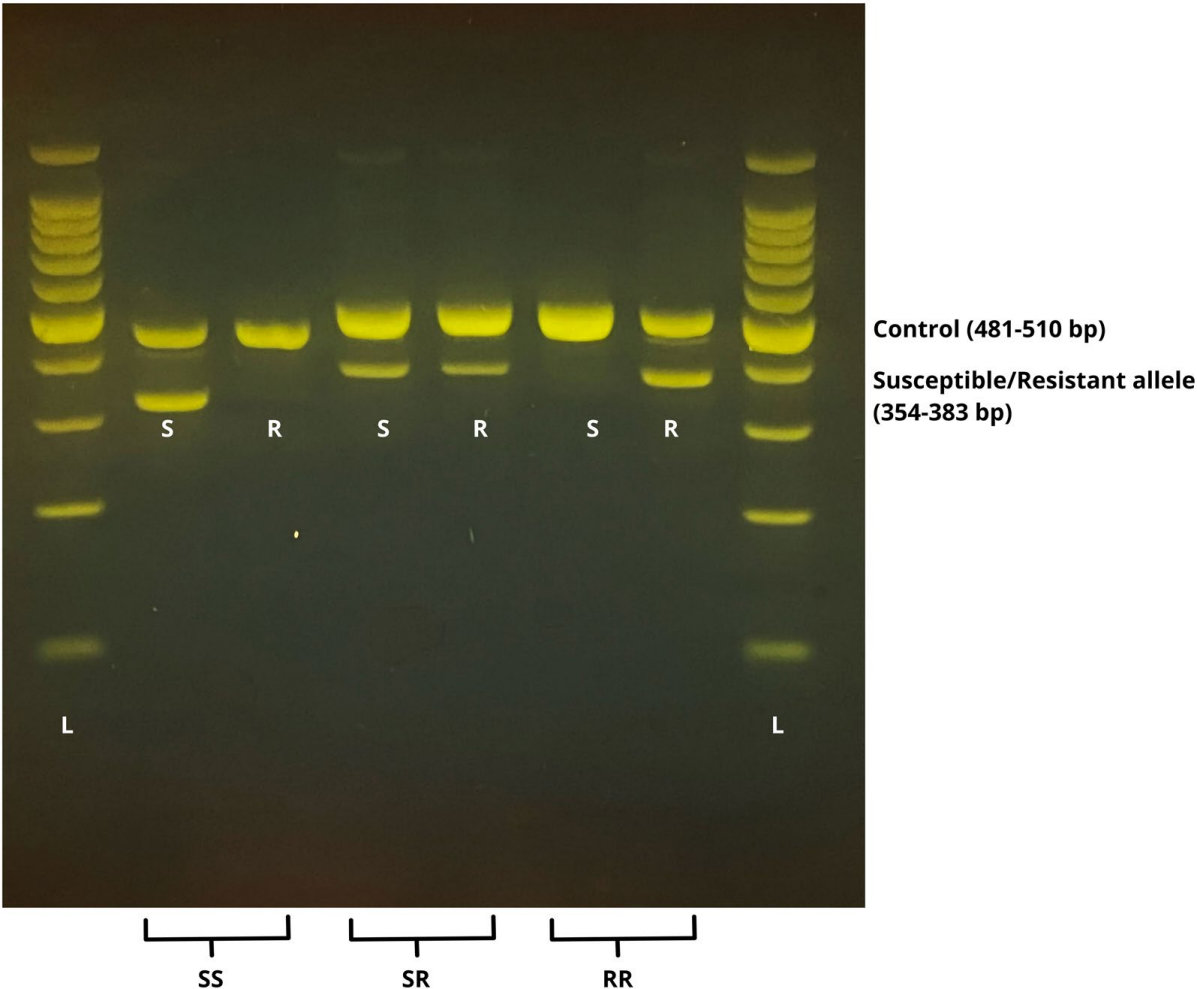
Representative electrophoretic profiles of the allele-specific diagnostic PCR assay for detecting the *kdr* mutation L1014F in *Culex pipiens*. In the case of each sample, two parallel PCR reactions were made with primers amplifying the control (481–510 bp), the susceptible (S), and/or the resistant (R) alleles (354-383 bp) from genomic DNA (L: 100 bp ladder (Promega, Wisconsin, USA), genotypes of homozygote susceptible (SS), heterozygote (SR), and homozygote resistant (RR) mosquitoes)

In *Culex* species, one of the most prevalent *kdr* point mutations associated with resistance in the voltage-sensitive sodium channel (*vssc*) gene is L1014F. For the detection of this mutation in *Cx. pipiens* specimens, we performed a quick, allele-specific polymerase chain reaction (PCR)-based diagnostic test described previously by Martinez-Torres et al. [35]. Briefly, two separate PCR reactions were run in parallel, using Cgd1 (GTGGAACTTCACCGACTTC), Cgd2 (GCAAGGCTAAGAAAAGGTTAAG), and Cgd3 (CCACCGTAGTGATAGGAAATTTA) primers to identify the presence of the wild-type susceptible allele (L1014), and the Cgd1, Cgd2, and Cgd4 (CCACCGTAGTGATAGGAAATTTT) primers to detect the leucine–phenylalanine substitution, i.e., to identify the presence of the resistant allele (F1014). For the PCR reactions, the HotStarTaq Master Mix Kit (QIAGEN, Hilden, Germany) was used, with the following parameters and conditions: 12.5 µL Master Mix, 7.5 µL 2 μM primer mix (contained an equal amount of each primer), 4 µL nuclease-free water, and 1 µL template per tube. The thermocycler conditions included an initial activation step at 95 °C for 15 min followed by 40 cycles of denaturation step at 94 °C for 30 s, an elongation step at 52 °C for 30 s, an extension step at 72 °C for 1 min, and a final extension at 72 °C for 10 min. The PCR amplicons were visualized by electrophoresis in a 1.5% agarose gel. The results of both reactions were used to determine the genotype of each individual as resistant homozygote (F/F: RR), resistant heterozygote (L/F: SR), or susceptible homozygote (L/L: SS) (Fig. 2).

### Detection of *kdr* mutations V1016G and F1534C in *Aedes albopictus*

Among the most prevalent mutations of the *vssc* gene responsible for pyrethroid resistance in *Ae. albopictus*, V1016G is the most widely investigated in Europe, while the F1534C mutation has been reported as the most common *kdr* mutation in Southeast Asia, in the native range of this species. Since the origin of the investigated populations of *Ae. albopictus* is unknown, we involved both codons in our analysis. For the detection of V1016G mutation, we partially modified the protocol described by Pichler et al. [36], since we ran two separate and parallel PCRs to detect the valine–glycine substitution, i.e., to identify the presence of resistant allele (G1016) of the *vssc* gene. Briefly, Albo1016F (5′ -AGTGCTGCGTGACCAACAGATCYGWACTAATCGGAGAATG-3′) with Gly1016R (5′-GCGGGCAGGGCGGCGGGGGCGGGGCCAGCAAGGCTAAGAAAAGGTTAACTC-3′), and Albo1016F with Val1016R (5′-GCGGGCAGCAAGGCTAAGAAAAGGTTAATTA-3′) primers were used, in parallel, using the HotStarTaq Master Mix Kit (QIAGEN, Hilden, Germany). Each reaction included 12.5 µL Master Mix, 2–2 µL 2 µM primers (F and R), 6.5 µL nuclease-free water, and 1 µL template. The thermocycler conditions included an initial heat activation at 95 °C for 15 min followed by 40 cycles of denaturation step at 94 °C for 30 s, an elongation step at 57 °C for 30 s, an extension step at 72 °C for 1 min, and a final extension at 10 min at 72 °C. For the detection of F1534C mutation, we followed the same methodology described above, using the primers F1534Phe (5′-GCG GGC TCT ACT TTG TGT TCT TCA TCA TAT T-3′) with 1534R (5′-TCT GCT CGT TGA AGT TGT CGA T-3′) and F1534Cys ^kdr^ (5′-GCG GGC AGG GCG GCG GGG GCG GGG CCT CTA CTT TGT GTT CTT CAT CAT GTG-3′) with 1534R [37]. The thermocycler conditions included an initial heat activation at 95 °C for 15 min followed by 35 cycles of denaturation step at 94 °C for 30 s, an elongation step at 55 °C for 30 s, an extension step at 72 °C for 1 min, and a final extension at 10 min at 72 °C. After the PCR, the amplicons were separated by electrophoresis on 4% agarose gel, allowing the visualization and determination of the genotype, similar to the case of genotyping *Cx. pipiens*.

## Results

Overall, 302 *Cx. pipiens* mosquito specimens were analyzed for the presence of the point mutation L1014F responsible for pyrethroid resistance. The mutation, i.e., resistance allele, was detected in all five counties involved in the study, at a frequency ranging from 18.1% to 36.3%, with the highest frequency detected in the population from Pest County. The overall (all study sites/mosquito samples) frequency of the resistance allele was recorded at 30.4% (Table 2). Resistance alleles were identified in homozygosity (24 out of the 302 investigated specimens) and in heterozygosity with the susceptible allele as well (136 out of the 302 investigated specimens), showing that 53% of the investigated *Cx. pipiens* mosquito individuals were resistant to pyrethroids. Although we were able to analyze a different number of specimens in each county, the homozygous susceptible genotype (L/L) was always detected with a frequency > 45% except for in Baranya County, where heterozygous genotype (L/F) showed the highest prevalence (52% of the investigated specimens) (Table 2).

**Table 2:** Genotype and allele frequencies of the L1014F *kdr* mutation in *Culex pipiens* populations from Hungary

County	No. of investigated mosquito individuals (*n*)	RR (*n*)	SR (*n*)	SS (*n*)	%R alleles
Győr-Moson-Sopron	100	7	46	47	30.0
Pest	26	5	9	12	36.3
Hajdú-Bihar	5	0	2	3	20.0
Baranya	124	11	64	49	34.7
Somogy	47	1	15	31	18.1
Total	302	24	136	142	30.4

RR: the number of mosquitoes homozygous for the resistant mutation, SR: the number of heterozygous mosquitoes holding both susceptible and resistant alleles, SS: the number of mosquitoes homozygous for the susceptible allele, %R alleles: frequency of the resistant allele in the investigated populations, evaluated according to Hardy–Weinberg equilibrium

In the case of *Ae. albopictus*, a total of 120 mosquito specimens were involved in the analysis to investigate the presence of V1016G and/or F1534C *kdr* mutation, responsible for pyrethroid resistance. Genotyping analyses revealed that all the specimens, regardless of the collection site, showed a homozygous susceptible (SS) genotype for both codons, indicating that no mosquitoes carrying the resistant alleles are present in the investigated populations.

## Discussion

In the field of pyrethroid resistance research, extensive studies have been conducted globally, particularly in regions where mosquito-borne diseases are endemic and mainly focused on mosquito species that serve as potential vectors. In Africa and Asia, significant resistance has been documented in *Anopheles* and *Aedes* species, posing challenges for malaria and dengue control programs [38–42]. In Europe, studies have primarily focused on *Cx. pipiens* and *Ae. albopictus* species, and typically evaluating the presence of *kdr* mutations, which are known to confer resistance to pyrethroids [36, 43–45, 49]. Although mosquitoes carrying resistance alleles have been detected in several countries in the last decades, there are still limited data available from the continent, especially from regions where chemical mosquito control is still dominant.

Here, we report the first research on pyrethroid resistance in mosquito populations from Hungary. Based on PCR genotyping, we detected the presence of the L1014F mutation in all the investigated field-collected populations of *Cx. pipiens*, with more than 50% of specimens being resistant to pyrethroids. Considering the mosquito control strategy of Hungary, where chemical treatments with deltamethrin are applied in approximately 90% of the controlled areas [31], this extensive use of insecticides could have contributed significantly to the development and spread of resistance within the populations of *Cx. pipiens*. The high reliance on chemical methods (extensive and frequent use of deltamethrin) increased selection pressure, leading to the widespread presence of resistant individuals. Similar results have been found in other European countries, including Italy, Spain, Belgium, Greece, Slovakia, and Romania, where *Culex pipiens* mosquitoes carry the resistant allele for pyrethroids with a high frequency (in both homozygosity and heterozygosity with the susceptible allele) [43–45]. Also, the species show phenotypic resistance to deltamethrin on the basis of the World Health Organization (WHO) bottle assay, a standard operating procedure for testing insecticide susceptibility of adult mosquitoes [46–48].

In the case of *Ae. albopictus*, studies on insecticide resistance have primarily focused on countries where the species has become well established and have shown varying levels of resistance across different regions. For instance, significant pyrethroid resistance has been observed in *Ae. albopictus* populations in Italy, particularly in urban areas with frequent insecticide use [36, 49]. In Spain, varying levels of susceptibility have been detected across different regions, as demonstrated by WHO bottle assays and biochemical tests [48, 50]. Furthermore, the study by Pichler et al. also suggests that pyrethroid resistance is emerging across Europe, including France, Switzerland, Romania, Bulgaria, and Turkey, as evidenced by the geographic distribution of the V1016G *kdr* mutation in European *Ae. albopictus* populations [51].

On the basis of our analysis, the genetic alleles responsible for resistance were not present in the Hungarian populations of *Ae. albopictus*, nor on 1016 or 1534 codons. Although the invasive species emerged in Europe more than 30 years ago and has been distributed across the continent since then [52], it has been recently introduced to Hungary, being first recorded in 2014 [30]. The origin of its introduction into the country and its genetic relation with other European populations have not yet been revealed, but during this relatively short period, it has had limited exposure to chemical insecticides in the frame of the mosquito control program. It is possible that the local population has not yet encountered the intense selection pressure required to drive the development of resistant traits. As a result, these populations may still be largely susceptible to pyrethroids. However, this also means that, if chemical control is not carefully managed, there is a risk that resistance could rapidly develop as the population is exposed to insecticides, such as in the case of *Cx. pipiens*. Therefore, these findings serve as a wake-up call for mosquito surveillance programs, underscoring the importance of including pyrethroid resistance monitoring in their activities [51].

Moreover, our results indicate an urgent need for a change of concept in mosquito control strategy in Hungary. There is no countrywide surveillance of mosquitoes or vector-borne diseases, and the current national mosquito control program focuses on the rapid eradication of mosquito populations, dominantly by chemical adulticide treatments. The IVM concept, mentioned above, along with limited (restricted) use of chemical control according to the EU standards, is crucial to prevent the emergence of resistance in *Ae. albopictus* populations in Hungary, and to decrease the public health risk of vector-borne diseases such as WNV. Even if the use of deltamethrin is halted soon, the *kdr* mutation will not disappear from the resistant *Cx. pipiens* populations probably for decades [53]. Therefore, it is crucial to act as soon as possible.

Our present study may be an important initial step toward sustainable mosquito control in Hungary, provided that our results reach and are effectively implemented by decision-makers. However, it is essential to continue these efforts with regular monitoring of pyrethroid resistance patterns in *Ae. albopictus* populations, as well as to get a whole picture of the situation in the country. It could be relevant to investigate further types of *vssc* mutations in the species at codon 1534 (F1534C, F1534S, F1534L, etc.) that can be related to pyrethroid resistance. Given that public awareness and community engagement are crucial elements of an effective strategy, it is important to educate the public about the importance of participating locally in mosquito control efforts, particularly by eliminating artificial breeding sites in private gardens. In Hungary, chemical control is often considered the most effective option by society owing to its long-established use and the lack of information. Therefore, public education is vital to introduce and promote alternative, sustainable solutions as viable options.

## Conclusions

Our study on pyrethroid resistance in *Cx. pipiens* and *Ae. albopictus* mosquitoes aims to fill the research gap in Hungary and is a major step toward more sustainable vector control. We revealed the presence of the *kdr* mutation responsible for pyrethroid resistance in *Cx. pipiens* mosquitoes, highlighting the consequences of the unilateral and long-term use of chemical treatments on native mosquito fauna. Moreover, this indicates an urgent need for a change of concept in the control strategy in countries where mosquito control still relies dominantly on chemical treatments. The moderate use of chemical control is highly recommended to prevent the development of pyrethroid resistance in recently established populations of the invasive *Ae. albopictus*, which has already shown the responsible *kdr* mutation in several European countries at codon 1016, although it could be convenient to investigate further mutations as well. The most severe consequence of emerging resistance is that, in the event of a vector-borne disease epidemic, chemical treatments—the only tool available to us to rapidly eliminate vectors—would become ineffective against resistant mosquito populations. Hence, these findings serve as a wake-up call for mosquito surveillance programs to address and incorporate resistance monitoring into their strategies.

## Supplementary Information

Additional file 1: Dataset S1. Data on field collection of *Culex pipiens* and *Aedes albopictus* mosquito specimens involved in the present analyses.

## Data Availability

No datasets were generated or analyzed during the current study.
