# Supplementary material for: Consequences of insecticide overuse in Hungary: assessment of pyrethroid resistance in Culex pipiens and Aedes albopictus mosquitoes
Source: Parasit Vectors. 2025 Jan 16;18:13. doi: 10.1186/s13071-024-06635-5 (PMC11740463; doi:10.1186/s13071-024-06635-5)
Supplement: Supplementary file 1 — Additional file 1: Dataset S1. Data on field collection of Culex pipiens and Aedes albopictus mosquito specimens involved in the present analyses. [file 13071_2024_6635_MOESM1_ESM.pdf]

Supplementary information

Additional file 1: Dataset S1. Data on field collection of *Culex pipiens* and *Aedes albopictus* mosquito specimens involved in the present analyses.

Tube code	Tube ID	Country	County	Municipality	Site code on Map	Longitude	Latitude	Collection year	Collection date	Trapping Method	Mosquito Species	DNA extraction kit	Pyrethroid resistance (L1014F, V1016G/F1534C)
HU-2022	86	Hungary	Baranya	Pécs	7	46.070833	18.233056	2022	2022.05.19.	EVS	*Culex pipiens*	Zymo - Quick-DNA Miniprep Plus Kit	Susceptible
HU-2022	303	Hungary	Baranya	Pécs	7	46.070833	18.233056	2022	2022.06.09.	EVS	*Culex pipiens*	Zymo - Quick-DNA Miniprep Plus Kit	Resistant
HU-2022	119	Hungary	Baranya	Pécs	7	46.070833	18.233056	2022	2022.06.16.	EVS	*Culex pipiens*	Zymo - Quick-DNA Miniprep Plus Kit	Susceptible
HU-2022	130	Hungary	Baranya	Pécs	7	46.070833	18.233056	2022	2022.06.16.	EVS	*Culex pipiens*	Zymo - Quick-DNA Miniprep Plus Kit	Susceptible
HU-2022	134	Hungary	Baranya	Pécs	7	46.070833	18.233056	2022	2022.06.16.	EVS	*Culex pipiens*	Zymo - Quick-DNA Miniprep Plus Kit	Susceptible
HU-2022	139	Hungary	Baranya	Pécs	7	46.070833	18.233056	2022	2022.06.23.	EVS	*Culex pipiens*	Zymo - Quick-DNA Miniprep Plus Kit	Susceptible
HU-2022	207	Hungary	Baranya	Pécs	7	46.070833	18.233056	2022	2022.06.30.	EVS	*Culex pipiens*	Zymo - Quick-DNA Miniprep Plus Kit	Susceptible
HU-2022	275	Hungary	Baranya	Pécs	7	46.070833	18.233056	2022	2022.07.07.	EVS	*Culex pipiens*	Zymo - Quick-DNA Miniprep Plus Kit	Heterozygous
HU-2022	379	Hungary	Baranya	Pécs	7	46.070833	18.233056	2022	2022.07.07.	EVS	*Culex pipiens*	Zymo - Quick-DNA Miniprep Plus Kit	Heterozygous
HU-2022	277	Hungary	Baranya	Pécs	7	46.070833	18.233056	2022	2022.07.07.	EVS	*Culex pipiens*	Zymo - Quick-DNA Miniprep Plus Kit	Susceptible
HU-2022	287	Hungary	Baranya	Pécs	7	46.070833	18.233056	2022	2022.07.21.	EVS	*Culex pipiens*	Zymo - Quick-DNA Miniprep Plus Kit	Susceptible
HU-2022	477	Hungary	Baranya	Pécs	7	46.070833	18.233056	2022	2022.07.28.	EVS	*Culex pipiens*	Zymo - Quick-DNA Miniprep Plus Kit	Heterozygous
HU-2022	38	Hungary	Baranya	Pécs	7	46.070833	18.233056	2022	2022.08.18.	EVS	*Culex pipiens*	Zymo - Quick-DNA Miniprep Plus Kit	Heterozygote
HU-2022	34	Hungary	Baranya	Pécs	7	46.070833	18.233056	2022	2022.08.18.	EVS	*Culex pipiens*	Zymo - Quick-DNA Miniprep Plus Kit	Susceptible
HU-2022	47	Hungary	Baranya	Pécs	7	46.070833	18.233056	2022	2022.08.25.	EVS	*Culex pipiens*	Zymo - Quick-DNA Miniprep Plus Kit	Susceptible
HU-2022	52	Hungary	Baranya	Pécs	7	46.070833	18.233056	2022	2022.08.26.	EVS	*Culex pipiens*	Zymo - Quick-DNA Miniprep Plus Kit	Heterozygous
HU-2022	58	Hungary	Baranya	Pécs	7	46.070833	18.233056	2022	2022.09.01.	EVS	*Culex pipiens*	Zymo - Quick-DNA Miniprep Plus Kit	Susceptible
HU-2022	59	Hungary	Baranya	Pécs	7	46.070833	18.233056	2022	2022.09.08.	EVS	*Culex pipiens*	Zymo - Quick-DNA Miniprep Plus Kit	Heterozygous
HU-2022	62	Hungary	Baranya	Pécs	7	46.070833	18.233056	2022	2022.09.08.	EVS	*Culex pipiens*	Zymo - Quick-DNA Miniprep Plus Kit	Resistant
HU-2022	74	Hungary	Baranya	Pécs	7	46.070833	18.233056	2022	2022.09.15.	EVS	*Culex pipiens*	Zymo - Quick-DNA Miniprep Plus Kit	Resistant
HU-2022	67	Hungary	Baranya	Pécs	7	46.070833	18.233056	2022	2022.09.15.	EVS	*Culex pipiens*	Zymo - Quick-DNA Miniprep Plus Kit	Susceptible
HU-2022	80	Hungary	Baranya	Pécs	7	46.070833	18.233056	2022	2022.09.22.	EVS	*Culex pipiens*	Zymo - Quick-DNA Miniprep Plus Kit	Heterozygous
HU-2022	75	Hungary	Baranya	Pécs	7	46.070833	18.233056	2022	2022.09.22.	EVS	*Culex pipiens*	Zymo - Quick-DNA Miniprep Plus Kit	Resistant

HU-2022	81	Hungary	Baranya	Pécs	7	46.070833	18.233056	2022	2022.09.22.	HLC	*Culex pipiens*	Zymo - Quick-DNA Miniprep Plus Kit	Susceptible
HU-2023	31	Hungary	Baranya	Pécs	7	46.070833	18.233056	2023	2023.05.10	EVS	*Culex pipiens*	Zymo - Quick-DNA Miniprep Plus Kit	Resistant
HU-2023	104	Hungary	Baranya	Pécs	7	46.070833	18.233056	2023	2023.05.24	EVS	*Culex pipiens*	Zymo - Quick-DNA Miniprep Plus Kit	Susceptible
HU-2023	165	Hungary	Baranya	Pécs	7	46.070833	18.233056	2023	2023.05.24	EVS	*Culex pipiens*	Zymo - Quick-DNA Miniprep Plus Kit	Susceptible
HU-2023	231	Hungary	Baranya	Pécs	7	46.070833	18.233056	2023	2023.06.01	EVS	*Culex pipiens*	Zymo - Quick-DNA Miniprep Plus Kit	Susceptible
HU-2023	235	Hungary	Baranya	Pécs	7	46.070833	18.233056	2023	2023.06.01	EVS	*Culex pipiens*	Zymo - Quick-DNA Miniprep Plus Kit	Susceptible
HU-2023	268	Hungary	Baranya	Pécs	7	46.070833	18.233056	2023	2023.06.01	EVS	*Culex pipiens*	Zymo - Quick-DNA Miniprep Plus Kit	Susceptible
HU-2023	308_1	Hungary	Baranya	Pécs	7	46.070833	18.233056	2023	2023.06.05.	EVS	*Culex pipiens*	Zymo - Quick-DNA Miniprep Plus Kit	Susceptible
HU-2023	433_1	Hungary	Baranya	Pécs	7	46.070833	18.233056	2023	2023.06.07	EVS	*Culex pipiens*	Zymo - Quick-DNA Miniprep Plus Kit	Heterozygous
HU-2023	424	Hungary	Baranya	Pécs	7	46.070833	18.233056	2023	2023.06.07	EVS	*Culex pipiens*	Zymo - Quick-DNA Miniprep Plus Kit	Susceptible
HU-2023	508_1	Hungary	Baranya	Pécs	7	46.070833	18.233056	2023	2023.06.07	EVS	*Culex pipiens*	Zymo - Quick-DNA Miniprep Plus Kit	Susceptible
HU-2023	508_2	Hungary	Baranya	Pécs	7	46.070833	18.233056	2023	2023.06.07	EVS	*Culex pipiens*	Zymo - Quick-DNA Miniprep Plus Kit	Susceptible
HU-2023	335_1	Hungary	Baranya	Pécs	7	46.070833	18.233056	2023	2023.06.07.	EVS	*Culex pipiens*	Zymo - Quick-DNA Miniprep Plus Kit	Heterozygous
HU-2023	360_1	Hungary	Baranya	Pécs	7	46.070833	18.233056	2023	2023.06.07.	EVS	*Culex pipiens*	Zymo - Quick-DNA Miniprep Plus Kit	Heterozygous
HU-2023	401_1	Hungary	Baranya	Pécs	7	46.070833	18.233056	2023	2023.06.07.	EVS	*Culex pipiens*	Zymo - Quick-DNA Miniprep Plus Kit	Heterozygous
HU-2023	620	Hungary	Baranya	Pécs	7	46.070833	18.233056	2023	2023.06.14	EVS	*Culex pipiens*	Zymo - Quick-DNA Miniprep Plus Kit	Resistant
HU-2023	604	Hungary	Baranya	Pécs	7	46.070833	18.233056	2023	2023.06.14	EVS	*Culex pipiens*	Zymo - Quick-DNA Miniprep Plus Kit	Susceptible
HU-2023	854_1	Hungary	Baranya	Pécs	7	46.070833	18.233056	2023	2023.06.22	EVS	*Culex pipiens*	Zymo - Quick-DNA Miniprep Plus Kit	Resistant
HU-2023	741_1	Hungary	Baranya	Pécs	7	46.070833	18.233056	2023	2023.06.22	EVS	*Culex pipiens*	Zymo - Quick-DNA Miniprep Plus Kit	Susceptible
HU-2023	814_1	Hungary	Baranya	Pécs	7	46.070833	18.233056	2023	2023.06.22.	EVS	*Culex pipiens*	Zymo - Quick-DNA Miniprep Plus Kit	Susceptible
HU-2023	865_1	Hungary	Baranya	Pécs	7	46.070833	18.233056	2023	2023.06.29	EVS	*Culex pipiens*	Zymo - Quick-DNA Miniprep Plus Kit	Susceptible
HU-2023	889_1	Hungary	Baranya	Pécs	7	46.070833	18.233056	2023	2023.06.29	EVS	*Culex pipiens*	Zymo - Quick-DNA Miniprep Plus Kit	Susceptible
HU-2023	918_1	Hungary	Baranya	Pécs	7	46.070833	18.233056	2023	2023.06.29	EVS	*Culex pipiens*	Zymo - Quick-DNA Miniprep Plus Kit	Susceptible
HU-2023	1010_1	Hungary	Baranya	Pécs	7	46.070833	18.233056	2023	2023.07.06	EVS	*Culex pipiens*	Zymo - Quick-DNA Miniprep Plus Kit	Heterozygous
HU-2023	981_1	Hungary	Baranya	Pécs	7	46.070833	18.233056	2023	2023.07.06	EVS	*Culex pipiens*	Zymo - Quick-DNA Miniprep Plus Kit	Heterozygous
HU-2023	993_1	Hungary	Baranya	Pécs	7	46.070833	18.233056	2023	2023.07.06	EVS	*Culex pipiens*	Zymo - Quick-DNA Miniprep Plus Kit	Heterozygous
HU-2023	993_2	Hungary	Baranya	Pécs	7	46.070833	18.233056	2023	2023.07.06	EVS	*Culex pipiens*	Zymo - Quick-DNA Miniprep Plus Kit	Heterozygous
HU-2023	961_1	Hungary	Baranya	Pécs	7	46.070833	18.233056	2023	2023.07.06	EVS	*Culex pipiens*	Zymo - Quick-DNA Miniprep Plus Kit	Susceptible

HU-2023	966_1	Hungary	Baranya	Pécs	7	46.070833	18.233056	2023	2023.07.06	EVS	*Culex pipiens*	Zymo - Quick-DNA Miniprep Plus Kit	Susceptible
HU-2023	1010_2	Hungary	Baranya	Pécs	7	46.070833	18.233056	2023	2023.07.06.	EVS	*Culex pipiens*	Zymo - Quick-DNA Miniprep Plus Kit	Susceptible
HU-2023	971_1	Hungary	Baranya	Pécs	7	46.070833	18.233056	2023	2023.07.06.	EVS	*Culex pipiens*	Zymo - Quick-DNA Miniprep Plus Kit	Susceptible
HU-2023	1050	Hungary	Baranya	Pécs	7	46.070833	18.233056	2023	2023.07.12	EVS	*Culex pipiens*	Zymo - Quick-DNA Miniprep Plus Kit	Heterozygous
HU-2023	1019_1	Hungary	Baranya	Pécs	7	46.070833	18.233056	2023	2023.07.12	EVS	*Culex pipiens*	Zymo - Quick-DNA Miniprep Plus Kit	Heterozygous
HU-2023	1022_1	Hungary	Baranya	Pécs	7	46.070833	18.233056	2023	2023.07.12	EVS	*Culex pipiens*	Zymo - Quick-DNA Miniprep Plus Kit	Heterozygous
HU-2023	1027_1	Hungary	Baranya	Pécs	7	46.070833	18.233056	2023	2023.07.12	EVS	*Culex pipiens*	Zymo - Quick-DNA Miniprep Plus Kit	Heterozygous
HU-2023	1027_2	Hungary	Baranya	Pécs	7	46.070833	18.233056	2023	2023.07.12	EVS	*Culex pipiens*	Zymo - Quick-DNA Miniprep Plus Kit	Heterozygous
HU-2023	1032_1	Hungary	Baranya	Pécs	7	46.070833	18.233056	2023	2023.07.12	EVS	*Culex pipiens*	Zymo - Quick-DNA Miniprep Plus Kit	Heterozygous
HU-2023	1032_2	Hungary	Baranya	Pécs	7	46.070833	18.233056	2023	2023.07.12	EVS	*Culex pipiens*	Zymo - Quick-DNA Miniprep Plus Kit	Heterozygous
HU-2023	1022_2	Hungary	Baranya	Pécs	7	46.070833	18.233056	2023	2023.07.12	EVS	*Culex pipiens*	Zymo - Quick-DNA Miniprep Plus Kit	Susceptible
HU-2023	1014_1	Hungary	Baranya	Pécs	7	46.070833	18.233056	2023	2023.07.12.	EVS	*Culex pipiens*	Zymo - Quick-DNA Miniprep Plus Kit	Heterozygous
HU-2023	1014_2	Hungary	Baranya	Pécs	7	46.070833	18.233056	2023	2023.07.12.	EVS	*Culex pipiens*	Zymo - Quick-DNA Miniprep Plus Kit	Heterozygous
HU-2023	1019_2	Hungary	Baranya	Pécs	7	46.070833	18.233056	2023	2023.07.12.	EVS	*Culex pipiens*	Zymo - Quick-DNA Miniprep Plus Kit	Heterozygous
HU-2023	1051_1	Hungary	Baranya	Pécs	7	46.070833	18.233056	2023	2023.07.19	EVS	*Culex pipiens*	Zymo - Quick-DNA Miniprep Plus Kit	Heterozygous
HU-2023	1051_2	Hungary	Baranya	Pécs	7	46.070833	18.233056	2023	2023.07.19	EVS	*Culex pipiens*	Zymo - Quick-DNA Miniprep Plus Kit	Heterozygous
HU-2023	1054_1	Hungary	Baranya	Pécs	7	46.070833	18.233056	2023	2023.07.19	EVS	*Culex pipiens*	Zymo - Quick-DNA Miniprep Plus Kit	Heterozygous
HU-2023	1054_2	Hungary	Baranya	Pécs	7	46.070833	18.233056	2023	2023.07.19	EVS	*Culex pipiens*	Zymo - Quick-DNA Miniprep Plus Kit	Heterozygous
HU-2023	1057_1	Hungary	Baranya	Pécs	7	46.070833	18.233056	2023	2023.07.19	EVS	*Culex pipiens*	Zymo - Quick-DNA Miniprep Plus Kit	Heterozygous
HU-2023	1057_2	Hungary	Baranya	Pécs	7	46.070833	18.233056	2023	2023.07.19	EVS	*Culex pipiens*	Zymo - Quick-DNA Miniprep Plus Kit	Heterozygous
HU-2023	1060_1	Hungary	Baranya	Pécs	7	46.070833	18.233056	2023	2023.07.19	EVS	*Culex pipiens*	Zymo - Quick-DNA Miniprep Plus Kit	Heterozygous
HU-2023	1060_2	Hungary	Baranya	Pécs	7	46.070833	18.233056	2023	2023.07.19	EVS	*Culex pipiens*	Zymo - Quick-DNA Miniprep Plus Kit	Heterozygous
HU-2023	1067_1	Hungary	Baranya	Pécs	7	46.070833	18.233056	2023	2023.07.19	EVS	*Culex pipiens*	Zymo - Quick-DNA Miniprep Plus Kit	Heterozygous
HU-2023	1067_2	Hungary	Baranya	Pécs	7	46.070833	18.233056	2023	2023.07.19	EVS	*Culex pipiens*	Zymo - Quick-DNA Miniprep Plus Kit	Heterozygous
HU-2023	1088	Hungary	Baranya	Pécs	7	46.070833	18.233056	2023	2023.07.26	EVS	*Culex pipiens*	Zymo - Quick-DNA Miniprep Plus Kit	Heterozygous
HU-2023	1083_1	Hungary	Baranya	Pécs	7	46.070833	18.233056	2023	2023.07.26	EVS	*Culex pipiens*	Zymo - Quick-DNA Miniprep Plus Kit	Heterozygous
HU-2023	1085_1	Hungary	Baranya	Pécs	7	46.070833	18.233056	2023	2023.07.26	EVS	*Culex pipiens*	Zymo - Quick-DNA Miniprep Plus Kit	Heterozygous
HU-2023	1090_1	Hungary	Baranya	Pécs	7	46.070833	18.233056	2023	2023.07.26	EVS	*Culex pipiens*	Zymo - Quick-DNA Miniprep Plus Kit	Heterozygous

HU-2023	1097_1	Hungary	Baranya	Pécs	7	46.070833	18.233056	2023	2023.07.26	EVS	*Culex pipiens*	Zymo - Quick-DNA Miniprep Plus Kit	Heterozygous
HU-2023	1103_1	Hungary	Baranya	Pécs	7	46.070833	18.233056	2023	2023.08.02.	EVS	*Culex pipiens*	Zymo - Quick-DNA Miniprep Plus Kit	Heterozygous
HU-2023	1104_1	Hungary	Baranya	Pécs	7	46.070833	18.233056	2023	2023.08.02.	EVS	*Culex pipiens*	Zymo - Quick-DNA Miniprep Plus Kit	Heterozygous
HU-2023	1106_1	Hungary	Baranya	Pécs	7	46.070833	18.233056	2023	2023.08.02.	EVS	*Culex pipiens*	Zymo - Quick-DNA Miniprep Plus Kit	Heterozygous
HU-2023	1123	Hungary	Baranya	Pécs	7	46.070833	18.233056	2023	2023.08.09	EVS	*Culex pipiens*	Zymo - Quick-DNA Miniprep Plus Kit	Heterozygous
HU-2023	1118_1	Hungary	Baranya	Pécs	7	46.070833	18.233056	2023	2023.08.09	EVS	*Culex pipiens*	Zymo - Quick-DNA Miniprep Plus Kit	Heterozygous
HU-2023	1121_1	Hungary	Baranya	Pécs	7	46.070833	18.233056	2023	2023.08.09	EVS	*Culex pipiens*	Zymo - Quick-DNA Miniprep Plus Kit	Heterozygous
HU-2023	1117	Hungary	Baranya	Pécs	7	46.070833	18.233056	2023	2023.08.09	EVS	*Culex pipiens*	Zymo - Quick-DNA Miniprep Plus Kit	Heterozygous
HU-2023	1132_1	Hungary	Baranya	Pécs	7	46.070833	18.233056	2023	2023.08.16	EVS	*Culex pipiens*	Zymo - Quick-DNA Miniprep Plus Kit	Heterozygous
HU-2023	1147	Hungary	Baranya	Pécs	7	46.070833	18.233056	2023	2023.08.23	EVS	*Culex pipiens*	Zymo - Quick-DNA Miniprep Plus Kit	Heterozygous
HU-2023	1159	Hungary	Baranya	Pécs	7	46.070833	18.233056	2023	2023.08.30	EVS	*Culex pipiens*	Zymo - Quick-DNA Miniprep Plus Kit	Heterozygous
HU-2023	1174	Hungary	Baranya	Pécs	7	46.070833	18.233056	2023	2023.08.30	EVS	*Culex pipiens*	Zymo - Quick-DNA Miniprep Plus Kit	Heterozygous
HU-2023	1167_1	Hungary	Baranya	Pécs	7	46.070833	18.233056	2023	2023.08.30	EVS	*Culex pipiens*	Zymo - Quick-DNA Miniprep Plus Kit	Heterozygous
HU-2023	1202	Hungary	Baranya	Pécs	7	46.070833	18.233056	2023	2023.08.30	EVS	*Culex pipiens*	Zymo - Quick-DNA Miniprep Plus Kit	Susceptible
HU-2023	1204	Hungary	Baranya	Pécs	7	46.070833	18.233056	2023	2023.09.06	EVS	*Culex pipiens*	Zymo - Quick-DNA Miniprep Plus Kit	Heterozygous
HU-2023	1214	Hungary	Baranya	Pécs	7	46.070833	18.233056	2023	2023.09.06	EVS	*Culex pipiens*	Zymo - Quick-DNA Miniprep Plus Kit	Heterozygous
HU-2023	1212_1	Hungary	Baranya	Pécs	7	46.070833	18.233056	2023	2023.09.06	EVS	*Culex pipiens*	Zymo - Quick-DNA Miniprep Plus Kit	Heterozygous
HU-2023	1211_1	Hungary	Baranya	Pécs	7	46.070833	18.233056	2023	2023.09.06	EVS	*Culex pipiens*	Zymo - Quick-DNA Miniprep Plus Kit	Resistant
HU-2023	1212_2	Hungary	Baranya	Pécs	7	46.070833	18.233056	2023	2023.09.06	EVS	*Culex pipiens*	Zymo - Quick-DNA Miniprep Plus Kit	Resistant
HU-2023	1211_2	Hungary	Baranya	Pécs	7	46.070833	18.233056	2023	2023.09.06	EVS	*Culex pipiens*	Zymo - Quick-DNA Miniprep Plus Kit	Susceptible
HU-2023	1217	Hungary	Baranya	Pécs	7	46.070833	18.233056	2023	2023.09.06	EVS	*Culex pipiens*	Zymo - Quick-DNA Miniprep Plus Kit	Susceptible
HU-2023	1225	Hungary	Baranya	Pécs	7	46.070833	18.233056	2023	2023.09.06	EVS	*Culex pipiens*	Zymo - Quick-DNA Miniprep Plus Kit	Susceptible
HU-2023	1233	Hungary	Baranya	Pécs	7	46.070833	18.233056	2023	2023.09.14	EVS	*Culex pipiens*	Zymo - Quick-DNA Miniprep Plus Kit	Heterozygous
HU-2023	1238	Hungary	Baranya	Pécs	7	46.070833	18.233056	2023	2023.09.14	EVS	*Culex pipiens*	Zymo - Quick-DNA Miniprep Plus Kit	Resistant
HU-2023	1231	Hungary	Baranya	Pécs	7	46.070833	18.233056	2023	2023.09.14	EVS	*Culex pipiens*	Zymo - Quick-DNA Miniprep Plus Kit	Susceptible
HU-2023	1232_1	Hungary	Baranya	Pécs	7	46.070833	18.233056	2023	2023.09.14	EVS	*Culex pipiens*	Zymo - Quick-DNA Miniprep Plus Kit	Susceptible
HU-2023	1232_2	Hungary	Baranya	Pécs	7	46.070833	18.233056	2023	2023.09.14	EVS	*Culex pipiens*	Zymo - Quick-DNA Miniprep Plus Kit	Susceptible
HU-2023	1236_1	Hungary	Baranya	Pécs	7	46.070833	18.233056	2023	2023.09.14	EVS	*Culex pipiens*	Zymo - Quick-DNA Miniprep Plus Kit	Susceptible

HU-2023	1236_2	Hungary	Baranya	Pécs	7	46.070833	18.233056	2023	2023.09.14	EVS	*Culex pipiens*	Zymo - Quick-DNA Miniprep Plus Kit	Susceptible
HU-2023	1247	Hungary	Baranya	Pécs	7	46.070833	18.233056	2023	2023.09.21	EVS	*Culex pipiens*	Zymo - Quick-DNA Miniprep Plus Kit	Heterozygous
HU-2023	1252	Hungary	Baranya	Pécs	7	46.070833	18.233056	2023	2023.09.21	EVS	*Culex pipiens*	Zymo - Quick-DNA Miniprep Plus Kit	Resistant
HU-2023	1256	Hungary	Baranya	Pécs	7	46.070833	18.233056	2023	2023.09.21	EVS	*Culex pipiens*	Zymo - Quick-DNA Miniprep Plus Kit	Susceptible
HU-2023	1259	Hungary	Baranya	Pécs	7	46.070833	18.233056	2023	2023.09.28	EVS	*Culex pipiens*	Zymo - Quick-DNA Miniprep Plus Kit	Heterozygous
HU-2023	1261	Hungary	Baranya	Pécs	7	46.070833	18.233056	2023	2023.09.28	EVS	*Culex pipiens*	Zymo - Quick-DNA Miniprep Plus Kit	Heterozygous
HU-2023	1265_1	Hungary	Baranya	Pécs	7	46.070833	18.233056	2023	2023.09.28	EVS	*Culex pipiens*	Zymo - Quick-DNA Miniprep Plus Kit	Heterozygous
HU-2023	1265_2	Hungary	Baranya	Pécs	7	46.070833	18.233056	2023	2023.09.28	EVS	*Culex pipiens*	Zymo - Quick-DNA Miniprep Plus Kit	Heterozygous
HU-2023	1257	Hungary	Baranya	Pécs	7	46.070833	18.233056	2023	2023.09.28	EVS	*Culex pipiens*	Zymo - Quick-DNA Miniprep Plus Kit	Susceptible
HU-2023	1268	Hungary	Baranya	Pécs	7	46.070833	18.233056	2023	2023.09.28	EVS	*Culex pipiens*	Zymo - Quick-DNA Miniprep Plus Kit	Susceptible
HU-2023	1265_3	Hungary	Baranya	Pécs	7	46.070833	18.233056	2023	2023.09.28	EVS	*Culex pipiens*	Zymo - Quick-DNA Miniprep Plus Kit	Susceptible
HU-2023	1270	Hungary	Baranya	Pécs	7	46.070833	18.233056	2023	2023.10.05.	EVS	*Culex pipiens*	Zymo - Quick-DNA Miniprep Plus Kit	Susceptible
HU-2023	1272_1	Hungary	Baranya	Pécs	7	46.070833	18.233056	2023	2023.10.05.	EVS	*Culex pipiens*	Zymo - Quick-DNA Miniprep Plus Kit	Susceptible
HU-2023	1288	Hungary	Baranya	Pécs	7	46.070833	18.233056	2023	2023.10.12.	EVS	*Culex pipiens*	Zymo - Quick-DNA Miniprep Plus Kit	Heterozygous
HU-2023	1286_1	Hungary	Baranya	Pécs	7	46.070833	18.233056	2023	2023.10.12.	EVS	*Culex pipiens*	Zymo - Quick-DNA Miniprep Plus Kit	Susceptible
HU-2023	1296	Hungary	Baranya	Pécs	7	46.070833	18.233056	2023	2023.10.19.	EVS	*Culex pipiens*	Zymo - Quick-DNA Miniprep Plus Kit	Heterozygous
HU-2023	1306_1	Hungary	Baranya	Pécs	7	46.070833	18.233056	2023	2023.10.26.	EVS	*Culex pipiens*	Zymo - Quick-DNA Miniprep Plus Kit	Heterozygous
GYMS	188	Hungary	Győr-Moson-Sopron	Dunaszeg	2	47.768389	17.541411	2023	2023.06.10	EVS	*Culex pipiens*	Zymo - Quick-DNA Miniprep Plus Kit	Heterozygous
GYMS	189	Hungary	Győr-Moson-Sopron	Dunaszeg	2	47.768389	17.541411	2023	2023.06.10	EVS	*Culex pipiens*	Zymo - Quick-DNA Miniprep Plus Kit	Susceptible
GYMS	190	Hungary	Győr-Moson-Sopron	Dunaszeg	2	47.768389	17.541411	2023	2023.06.10	EVS	*Culex pipiens*	Zymo - Quick-DNA Miniprep Plus Kit	Susceptible
GYMS	191	Hungary	Győr-Moson-Sopron	Dunaszeg	2	47.768389	17.541411	2023	2023.06.10	EVS	*Culex pipiens*	Zymo - Quick-DNA Miniprep Plus Kit	Susceptible
GYMS	331	Hungary	Győr-Moson-Sopron	Dunaszeg	2	47.768389	17.541411	2023	2023.06.17	EVS	*Culex pipiens*	Zymo - Quick-DNA Miniprep Plus Kit	Heterozygous
GYMS	333	Hungary	Győr-Moson-Sopron	Dunaszeg	2	47.768389	17.541411	2023	2023.06.17	EVS	*Culex pipiens*	Zymo - Quick-DNA Miniprep Plus Kit	Heterozygous
GYMS	336	Hungary	Győr-Moson-Sopron	Dunaszeg	2	47.768389	17.541411	2023	2023.06.17	EVS	*Culex pipiens*	Zymo - Quick-DNA Miniprep Plus Kit	Heterozygous

GYMS	334	Hungary	Győr-Moson-Sopron	Dunaszeg	2	47.768389	17.541411	2023	2023.06.17	EVS	*Culex pipiens*	Zymo - Quick-DNA Miniprep Plus Kit	Resistant
GYMS	335	Hungary	Győr-Moson-Sopron	Dunaszeg	2	47.768389	17.541411	2023	2023.06.17	EVS	*Culex pipiens*	Zymo - Quick-DNA Miniprep Plus Kit	Resistant
GYMS	332	Hungary	Győr-Moson-Sopron	Dunaszeg	2	47.768389	17.541411	2023	2023.06.17	EVS	*Culex pipiens*	Zymo - Quick-DNA Miniprep Plus Kit	Susceptible
GYMS	337	Hungary	Győr-Moson-Sopron	Dunaszeg	2	47.768389	17.541411	2023	2023.06.17	EVS	*Culex pipiens*	Zymo - Quick-DNA Miniprep Plus Kit	Susceptible
GYMS	338	Hungary	Győr-Moson-Sopron	Dunaszeg	2	47.768389	17.541411	2023	2023.06.17	EVS	*Culex pipiens*	Zymo - Quick-DNA Miniprep Plus Kit	Susceptible
GYMS	339	Hungary	Győr-Moson-Sopron	Dunaszeg	2	47.768389	17.541411	2023	2023.06.17	EVS	*Culex pipiens*	Zymo - Quick-DNA Miniprep Plus Kit	Susceptible
GYMS	340	Hungary	Győr-Moson-Sopron	Dunaszeg	2	47.768389	17.541411	2023	2023.06.17	EVS	*Culex pipiens*	Zymo - Quick-DNA Miniprep Plus Kit	Susceptible
GYMS	341	Hungary	Győr-Moson-Sopron	Dunaszeg	2	47.768389	17.541411	2023	2023.06.17	EVS	*Culex pipiens*	Zymo - Quick-DNA Miniprep Plus Kit	Susceptible
GYMS	447	Hungary	Győr-Moson-Sopron	Dunaszeg	2	47.768389	17.541411	2023	2023.07.22	EVS	*Culex pipiens*	Zymo - Quick-DNA Miniprep Plus Kit	Heterozygous
GYMS	448	Hungary	Győr-Moson-Sopron	Dunaszeg	2	47.768389	17.541411	2023	2023.07.22	EVS	*Culex pipiens*	Zymo - Quick-DNA Miniprep Plus Kit	Heterozygous
GYMS	449	Hungary	Győr-Moson-Sopron	Dunaszeg	2	47.768389	17.541411	2023	2023.07.22	EVS	*Culex pipiens*	Zymo - Quick-DNA Miniprep Plus Kit	Heterozygous
GYMS	446	Hungary	Győr-Moson-Sopron	Dunaszeg	2	47.768389	17.541411	2023	2023.07.22	EVS	*Culex pipiens*	Zymo - Quick-DNA Miniprep Plus Kit	Susceptible
GYMS	492	Hungary	Győr-Moson-Sopron	Dunaszeg	2	47.768389	17.541411	2023	2023.10.07	EVS	*Culex pipiens*	Zymo - Quick-DNA Miniprep Plus Kit	Susceptible
GYMS	222	Hungary	Győr-Moson-Sopron	Hédervár	1	47.832431	17.456039	2023	2023.06.10	EVS	*Culex pipiens*	Zymo - Quick-DNA Miniprep Plus Kit	Heterozygous
GYMS	221	Hungary	Győr-Moson-Sopron	Hédervár	1	47.832431	17.456039	2023	2023.06.10	EVS	*Culex pipiens*	Zymo - Quick-DNA Miniprep Plus Kit	Susceptible
GYMS	362	Hungary	Győr-Moson-Sopron	Hédervár	1	47.832431	17.456039	2023	2023.06.17	EVS	*Culex pipiens*	Zymo - Quick-DNA Miniprep Plus Kit	Heterozygous
GYMS	363	Hungary	Győr-Moson-Sopron	Hédervár	1	47.832431	17.456039	2023	2023.06.17	EVS	*Culex pipiens*	Zymo - Quick-DNA Miniprep Plus Kit	Susceptible

GYMS	364	Hungary	Győr-Moson-Sopron	Hédervár	1	47.832431	17.456039	2023	2023.06.17	EVS	*Culex pipiens*	Zymo - Quick-DNA Miniprep Plus Kit	Susceptible
GYMS	365	Hungary	Győr-Moson-Sopron	Hédervár	1	47.832431	17.456039	2023	2023.06.17	EVS	*Culex pipiens*	Zymo - Quick-DNA Miniprep Plus Kit	Susceptible
GYMS	366	Hungary	Győr-Moson-Sopron	Hédervár	1	47.832431	17.456039	2023	2023.06.17	EVS	*Culex pipiens*	Zymo - Quick-DNA Miniprep Plus Kit	Susceptible
GYMS	367	Hungary	Győr-Moson-Sopron	Hédervár	1	47.832431	17.456039	2023	2023.06.17	EVS	*Culex pipiens*	Zymo - Quick-DNA Miniprep Plus Kit	Susceptible
GYMS	368	Hungary	Győr-Moson-Sopron	Hédervár	1	47.832431	17.456039	2023	2023.06.17	EVS	*Culex pipiens*	Zymo - Quick-DNA Miniprep Plus Kit	Susceptible
GYMS	369	Hungary	Győr-Moson-Sopron	Hédervár	1	47.832431	17.456039	2023	2023.06.17	EVS	*Culex pipiens*	Zymo - Quick-DNA Miniprep Plus Kit	Susceptible
GYMS	370	Hungary	Győr-Moson-Sopron	Hédervár	1	47.832431	17.456039	2023	2023.06.17	EVS	*Culex pipiens*	Zymo - Quick-DNA Miniprep Plus Kit	Susceptible
GYMS	371	Hungary	Győr-Moson-Sopron	Hédervár	1	47.832431	17.456039	2023	2023.06.17	EVS	*Culex pipiens*	Zymo - Quick-DNA Miniprep Plus Kit	Susceptible
GYMS	372	Hungary	Győr-Moson-Sopron	Hédervár	1	47.832431	17.456039	2023	2023.06.17	EVS	*Culex pipiens*	Zymo - Quick-DNA Miniprep Plus Kit	Susceptible
GYMS	479	Hungary	Győr-Moson-Sopron	Hédervár	1	47.832431	17.456039	2023	2023.07.22	EVS	*Culex pipiens*	Zymo - Quick-DNA Miniprep Plus Kit	Heterozygous
GYMS	481	Hungary	Győr-Moson-Sopron	Hédervár	1	47.832431	17.456039	2023	2023.07.22	EVS	*Culex pipiens*	Zymo - Quick-DNA Miniprep Plus Kit	Heterozygous
GYMS	478	Hungary	Győr-Moson-Sopron	Hédervár	1	47.832431	17.456039	2023	2023.07.22	EVS	*Culex pipiens*	Zymo - Quick-DNA Miniprep Plus Kit	Susceptible
GYMS	484	Hungary	Győr-Moson-Sopron	Hédervár	1	47.832431	17.456039	2023	2023.07.22	EVS	*Culex pipiens*	Zymo - Quick-DNA Miniprep Plus Kit	Susceptible
GYMS	230	Hungary	Győr-Moson-Sopron	Kisbajcs	3	47.744761	17.67975	2023	2023.06.10	EVS	*Culex pipiens*	Zymo - Quick-DNA Miniprep Plus Kit	Heterozygous
GYMS	231	Hungary	Győr-Moson-Sopron	Kisbajcs	3	47.744761	17.67975	2023	2023.06.10	EVS	*Culex pipiens*	Zymo - Quick-DNA Miniprep Plus Kit	Heterozygous
GYMS	233	Hungary	Győr-Moson-Sopron	Kisbajcs	3	47.744761	17.67975	2023	2023.06.10	EVS	*Culex pipiens*	Zymo - Quick-DNA Miniprep Plus Kit	Heterozygous
GYMS	229	Hungary	Győr-Moson-Sopron	Kisbajcs	3	47.744761	17.67975	2023	2023.06.10	EVS	*Culex pipiens*	Zymo - Quick-DNA Miniprep Plus Kit	Susceptible

GYMS	232	Hungary	Győr-Moson-Sopron	Kisbajcs	3	47.744761	17.67975	2023	2023.06.10	EVS	*Culex pipiens*	Zymo - Quick-DNA Miniprep Plus Kit	Susceptible
GYMS	234	Hungary	Győr-Moson-Sopron	Kisbajcs	3	47.744761	17.67975	2023	2023.06.10	EVS	*Culex pipiens*	Zymo - Quick-DNA Miniprep Plus Kit	Susceptible
GYMS	235	Hungary	Győr-Moson-Sopron	Kisbajcs	3	47.744761	17.67975	2023	2023.06.10	EVS	*Culex pipiens*	Zymo - Quick-DNA Miniprep Plus Kit	Susceptible
GYMS	236	Hungary	Győr-Moson-Sopron	Kisbajcs	3	47.744761	17.67975	2023	2023.06.10	EVS	*Culex pipiens*	Zymo - Quick-DNA Miniprep Plus Kit	Susceptible
GYMS	240	Hungary	Győr-Moson-Sopron	Kisbajcs	3	47.744761	17.67975	2023	2023.06.10	EVS	*Culex pipiens*	Zymo - Quick-DNA Miniprep Plus Kit	Susceptible
GYMS	241	Hungary	Győr-Moson-Sopron	Kisbajcs	3	47.744761	17.67975	2023	2023.06.10	EVS	*Culex pipiens*	Zymo - Quick-DNA Miniprep Plus Kit	Susceptible
GYMS	274	Hungary	Győr-Moson-Sopron	Kisbajcs	3	47.744761	17.67975	2023	2023.06.17	EVS	*Culex pipiens*	Zymo - Quick-DNA Miniprep Plus Kit	Heterozygous
GYMS	275	Hungary	Győr-Moson-Sopron	Kisbajcs	3	47.744761	17.67975	2023	2023.06.17	EVS	*Culex pipiens*	Zymo - Quick-DNA Miniprep Plus Kit	Heterozygous
GYMS	292	Hungary	Győr-Moson-Sopron	Kisbajcs	3	47.744761	17.67975	2023	2023.06.17	EVS	*Culex pipiens*	Zymo - Quick-DNA Miniprep Plus Kit	Heterozygous
GYMS	296	Hungary	Győr-Moson-Sopron	Kisbajcs	3	47.744761	17.67975	2023	2023.06.17	EVS	*Culex pipiens*	Zymo - Quick-DNA Miniprep Plus Kit	Heterozygous
GYMS	299	Hungary	Győr-Moson-Sopron	Kisbajcs	3	47.744761	17.67975	2023	2023.06.17	EVS	*Culex pipiens*	Zymo - Quick-DNA Miniprep Plus Kit	Heterozygous
GYMS	300	Hungary	Győr-Moson-Sopron	Kisbajcs	3	47.744761	17.67975	2023	2023.06.17	EVS	*Culex pipiens*	Zymo - Quick-DNA Miniprep Plus Kit	Heterozygous
GYMS	304	Hungary	Győr-Moson-Sopron	Kisbajcs	3	47.744761	17.67975	2023	2023.06.17	EVS	*Culex pipiens*	Zymo - Quick-DNA Miniprep Plus Kit	Heterozygous
GYMS	305	Hungary	Győr-Moson-Sopron	Kisbajcs	3	47.744761	17.67975	2023	2023.06.17	EVS	*Culex pipiens*	Zymo - Quick-DNA Miniprep Plus Kit	Heterozygous
GYMS	306	Hungary	Győr-Moson-Sopron	Kisbajcs	3	47.744761	17.67975	2023	2023.06.17	EVS	*Culex pipiens*	Zymo - Quick-DNA Miniprep Plus Kit	Heterozygous
GYMS	307	Hungary	Győr-Moson-Sopron	Kisbajcs	3	47.744761	17.67975	2023	2023.06.17	EVS	*Culex pipiens*	Zymo - Quick-DNA Miniprep Plus Kit	Heterozygous
GYMS	308	Hungary	Győr-Moson-Sopron	Kisbajcs	3	47.744761	17.67975	2023	2023.06.17	EVS	*Culex pipiens*	Zymo - Quick-DNA Miniprep Plus Kit	Heterozygous

GYMS	309	Hungary	Győr-Moson-Sopron	Kisbajcs	3	47.744761	17.67975	2023	2023.06.17	EVS	*Culex pipiens*	Zymo - Quick-DNA Miniprep Plus Kit	Heterozygous
GYMS	310	Hungary	Győr-Moson-Sopron	Kisbajcs	3	47.744761	17.67975	2023	2023.06.17	EVS	*Culex pipiens*	Zymo - Quick-DNA Miniprep Plus Kit	Heterozygous
GYMS	311	Hungary	Győr-Moson-Sopron	Kisbajcs	3	47.744761	17.67975	2023	2023.06.17	EVS	*Culex pipiens*	Zymo - Quick-DNA Miniprep Plus Kit	Heterozygous
GYMS	312	Hungary	Győr-Moson-Sopron	Kisbajcs	3	47.744761	17.67975	2023	2023.06.17	EVS	*Culex pipiens*	Zymo - Quick-DNA Miniprep Plus Kit	Heterozygous
GYMS	313	Hungary	Győr-Moson-Sopron	Kisbajcs	3	47.744761	17.67975	2023	2023.06.17	EVS	*Culex pipiens*	Zymo - Quick-DNA Miniprep Plus Kit	Heterozygous
GYMS	314	Hungary	Győr-Moson-Sopron	Kisbajcs	3	47.744761	17.67975	2023	2023.06.17	EVS	*Culex pipiens*	Zymo - Quick-DNA Miniprep Plus Kit	Heterozygous
GYMS	315	Hungary	Győr-Moson-Sopron	Kisbajcs	3	47.744761	17.67975	2023	2023.06.17	EVS	*Culex pipiens*	Zymo - Quick-DNA Miniprep Plus Kit	Heterozygous
GYMS	317	Hungary	Győr-Moson-Sopron	Kisbajcs	3	47.744761	17.67975	2023	2023.06.17	EVS	*Culex pipiens*	Zymo - Quick-DNA Miniprep Plus Kit	Heterozygous
GYMS	318	Hungary	Győr-Moson-Sopron	Kisbajcs	3	47.744761	17.67975	2023	2023.06.17	EVS	*Culex pipiens*	Zymo - Quick-DNA Miniprep Plus Kit	Heterozygous
GYMS	320	Hungary	Győr-Moson-Sopron	Kisbajcs	3	47.744761	17.67975	2023	2023.06.17	EVS	*Culex pipiens*	Zymo - Quick-DNA Miniprep Plus Kit	Heterozygous
GYMS	321	Hungary	Győr-Moson-Sopron	Kisbajcs	3	47.744761	17.67975	2023	2023.06.17	EVS	*Culex pipiens*	Zymo - Quick-DNA Miniprep Plus Kit	Heterozygous
GYMS	322	Hungary	Győr-Moson-Sopron	Kisbajcs	3	47.744761	17.67975	2023	2023.06.17	EVS	*Culex pipiens*	Zymo - Quick-DNA Miniprep Plus Kit	Heterozygous
GYMS	323	Hungary	Győr-Moson-Sopron	Kisbajcs	3	47.744761	17.67975	2023	2023.06.17	EVS	*Culex pipiens*	Zymo - Quick-DNA Miniprep Plus Kit	Heterozygous
GYMS	325	Hungary	Győr-Moson-Sopron	Kisbajcs	3	47.744761	17.67975	2023	2023.06.17	EVS	*Culex pipiens*	Zymo - Quick-DNA Miniprep Plus Kit	Heterozygous
GYMS	326	Hungary	Győr-Moson-Sopron	Kisbajcs	3	47.744761	17.67975	2023	2023.06.17	EVS	*Culex pipiens*	Zymo - Quick-DNA Miniprep Plus Kit	Heterozygous
GYMS	294	Hungary	Győr-Moson-Sopron	Kisbajcs	3	47.744761	17.67975	2023	2023.06.17	EVS	*Culex pipiens*	Zymo - Quick-DNA Miniprep Plus Kit	Resistant
GYMS	303	Hungary	Győr-Moson-Sopron	Kisbajcs	3	47.744761	17.67975	2023	2023.06.17	EVS	*Culex pipiens*	Zymo - Quick-DNA Miniprep Plus Kit	Resistant

GYMS	291	Hungary	Győr-Moson-Sopron	Kisbajcs	3	47.744761	17.67975	2023	2023.06.17	EVS	*Culex pipiens*	Zymo - Quick-DNA Miniprep Plus Kit	Susceptible
GYMS	293	Hungary	Győr-Moson-Sopron	Kisbajcs	3	47.744761	17.67975	2023	2023.06.17	EVS	*Culex pipiens*	Zymo - Quick-DNA Miniprep Plus Kit	Susceptible
GYMS	295	Hungary	Győr-Moson-Sopron	Kisbajcs	3	47.744761	17.67975	2023	2023.06.17	EVS	*Culex pipiens*	Zymo - Quick-DNA Miniprep Plus Kit	Susceptible
GYMS	297	Hungary	Győr-Moson-Sopron	Kisbajcs	3	47.744761	17.67975	2023	2023.06.17	EVS	*Culex pipiens*	Zymo - Quick-DNA Miniprep Plus Kit	Susceptible
GYMS	298	Hungary	Győr-Moson-Sopron	Kisbajcs	3	47.744761	17.67975	2023	2023.06.17	EVS	*Culex pipiens*	Zymo - Quick-DNA Miniprep Plus Kit	Susceptible
GYMS	301	Hungary	Győr-Moson-Sopron	Kisbajcs	3	47.744761	17.67975	2023	2023.06.17	EVS	*Culex pipiens*	Zymo - Quick-DNA Miniprep Plus Kit	Susceptible
GYMS	302	Hungary	Győr-Moson-Sopron	Kisbajcs	3	47.744761	17.67975	2023	2023.06.17	EVS	*Culex pipiens*	Zymo - Quick-DNA Miniprep Plus Kit	Susceptible
GYMS	316	Hungary	Győr-Moson-Sopron	Kisbajcs	3	47.744761	17.67975	2023	2023.06.17	EVS	*Culex pipiens*	Zymo - Quick-DNA Miniprep Plus Kit	Susceptible
GYMS	319	Hungary	Győr-Moson-Sopron	Kisbajcs	3	47.744761	17.67975	2023	2023.06.17	EVS	*Culex pipiens*	Zymo - Quick-DNA Miniprep Plus Kit	Susceptible
GYMS	324	Hungary	Győr-Moson-Sopron	Kisbajcs	3	47.744761	17.67975	2023	2023.06.17	EVS	*Culex pipiens*	Zymo - Quick-DNA Miniprep Plus Kit	Susceptible
GYMS	454	Hungary	Győr-Moson-Sopron	Kisbajcs	3	47.744761	17.67975	2023	2023.07.22	EVS	*Culex pipiens*	Zymo - Quick-DNA Miniprep Plus Kit	Heterozygous
GYMS	455	Hungary	Győr-Moson-Sopron	Kisbajcs	3	47.744761	17.67975	2023	2023.07.22	EVS	*Culex pipiens*	Zymo - Quick-DNA Miniprep Plus Kit	Heterozygous
GYMS	458	Hungary	Győr-Moson-Sopron	Kisbajcs	3	47.744761	17.67975	2023	2023.07.22	EVS	*Culex pipiens*	Zymo - Quick-DNA Miniprep Plus Kit	Heterozygous
GYMS	459	Hungary	Győr-Moson-Sopron	Kisbajcs	3	47.744761	17.67975	2023	2023.07.22	EVS	*Culex pipiens*	Zymo - Quick-DNA Miniprep Plus Kit	Heterozygous
GYMS	460	Hungary	Győr-Moson-Sopron	Kisbajcs	3	47.744761	17.67975	2023	2023.07.22	EVS	*Culex pipiens*	Zymo - Quick-DNA Miniprep Plus Kit	Heterozygous
GYMS	465	Hungary	Győr-Moson-Sopron	Kisbajcs	3	47.744761	17.67975	2023	2023.07.22	EVS	*Culex pipiens*	Zymo - Quick-DNA Miniprep Plus Kit	Heterozygous
GYMS	466	Hungary	Győr-Moson-Sopron	Kisbajcs	3	47.744761	17.67975	2023	2023.07.22	EVS	*Culex pipiens*	Zymo - Quick-DNA Miniprep Plus Kit	Heterozygous

GYMS	452	Hungary	Győr-Moson-Sopron	Kisbajcs	3	47.744761	17.67975	2023	2023.07.22	EVS	*Culex pipiens*	Zymo - Quick-DNA Miniprep Plus Kit	Resistant
GYMS	457	Hungary	Győr-Moson-Sopron	Kisbajcs	3	47.744761	17.67975	2023	2023.07.22	EVS	*Culex pipiens*	Zymo - Quick-DNA Miniprep Plus Kit	Resistant
GYMS	470	Hungary	Győr-Moson-Sopron	Kisbajcs	3	47.744761	17.67975	2023	2023.07.22	EVS	*Culex pipiens*	Zymo - Quick-DNA Miniprep Plus Kit	Resistant
GYMS	453	Hungary	Győr-Moson-Sopron	Kisbajcs	3	47.744761	17.67975	2023	2023.07.22	EVS	*Culex pipiens*	Zymo - Quick-DNA Miniprep Plus Kit	Susceptible
GYMS	456	Hungary	Győr-Moson-Sopron	Kisbajcs	3	47.744761	17.67975	2023	2023.07.22	EVS	*Culex pipiens*	Zymo - Quick-DNA Miniprep Plus Kit	Susceptible
GYMS	467	Hungary	Győr-Moson-Sopron	Kisbajcs	3	47.744761	17.67975	2023	2023.07.22	EVS	*Culex pipiens*	Zymo - Quick-DNA Miniprep Plus Kit	Susceptible
GYMS	468	Hungary	Győr-Moson-Sopron	Kisbajcs	3	47.744761	17.67975	2023	2023.07.22	EVS	*Culex pipiens*	Zymo - Quick-DNA Miniprep Plus Kit	Susceptible
GYMS	469	Hungary	Győr-Moson-Sopron	Kisbajcs	3	47.744761	17.67975	2023	2023.07.22	EVS	*Culex pipiens*	Zymo - Quick-DNA Miniprep Plus Kit	Susceptible
SZ_2022	164	Hungary	Hajdú-Bihar	Debrecen	6	47.53	21.639167	2022	2022.06.20	BG-Sentinel	*Culex pipiens*	Qiagen - QIAamp Viral RNA Mini Kit	Susceptible
SZ_2022	247	Hungary	Hajdú-Bihar	Debrecen	6	47.53	21.639167	2022	2022.07.04	BG-Sentinel	*Culex pipiens*	Qiagen - QIAamp Viral RNA Mini Kit	Heterozygous
SZ_2022	575	Hungary	Hajdú-Bihar	Debrecen	6	47.53	21.639167	2022	2022.08.14	BG-Sentinel	*Culex pipiens*	Qiagen - QIAamp Viral RNA Mini Kit	Susceptible
SZ_2022	987	Hungary	Hajdú-Bihar	Debrecen	6	47.53	21.639167	2022	2022.08.21	BG-Sentinel	*Culex pipiens*	Qiagen - QIAamp Viral RNA Mini Kit	Susceptible
SZ_2022	1018	Hungary	Hajdú-Bihar	Debrecen	6	47.53	21.639167	2022	2022.09.11	BG-Sentinel	*Culex pipiens*	Qiagen - QIAamp Viral RNA Mini Kit	Heterozygous
SZ_2022	138	Hungary	Pest	Budapest	4	47.498333	19.040833	2022	2022.06.20	BG-Sentinel	*Culex pipiens*	Qiagen - QIAamp Viral RNA Mini Kit	Heterozygous
SZ_2022	147	Hungary	Pest	Budapest	4	47.498333	19.040833	2022	2022.06.27	BG-Sentinel	*Culex pipiens*	Qiagen - QIAamp Viral RNA Mini Kit	Resistant
SZ_2022	468	Hungary	Pest	Budapest	4	47.498333	19.040833	2022	2022.07.04	BG-Sentinel	*Culex pipiens*	Qiagen - QIAamp Viral RNA Mini Kit	Heterozygous
SZ_2022	488	Hungary	Pest	Budapest	4	47.498333	19.040833	2022	2022.07.18	BG-Sentinel	*Culex pipiens*	Qiagen - QIAamp Viral RNA Mini Kit	Susceptible
SZ_2022	800	Hungary	Pest	Budapest	4	47.498333	19.040833	2022	2022.07.25	BG-Sentinel	*Culex pipiens*	Qiagen - QIAamp Viral RNA Mini Kit	Susceptible
SZ_2022	850	Hungary	Pest	Budapest	4	47.498333	19.040833	2022	2022.07.25	BG-Sentinel	*Culex pipiens*	Qiagen - QIAamp Viral RNA Mini Kit	Susceptible
SZ_2022	786	Hungary	Pest	Budapest	4	47.498333	19.040833	2022	2022.07.29	BG-Sentinel	*Culex pipiens*	Qiagen - QIAamp Viral RNA Mini Kit	Susceptible
SZ_2022	859	Hungary	Pest	Budapest	4	47.498333	19.040833	2022	2022.08.08	BG-Sentinel	*Culex pipiens*	Qiagen - QIAamp Viral RNA Mini Kit	Susceptible
SZ_2022	892	Hungary	Pest	Budapest	4	47.498333	19.040833	2022	2022.08.22	BG-Sentinel	*Culex pipiens*	Qiagen - QIAamp Viral RNA Mini Kit	Heterozygous
SZ_2022	910	Hungary	Pest	Budapest	4	47.498333	19.040833	2022	2022.08.29.	BG-Sentinel	*Culex pipiens*	Qiagen - QIAamp Viral RNA Mini Kit	Susceptible

SZ_2022	1187	Hungary	Pest	Budapest	4	47.498333	19.040833	2022	2022.10.03.	BG-Sentinel	*Culex pipiens*	Qiagen - QIAamp Viral RNA Mini Kit	Resistant
SZ_2023	39	Hungary	Pest	Budapest	4	47.498333	19.040833	2023	2023.06.23	BG-Sentinel	*Culex pipiens*	Qiagen - QIAamp Viral RNA Mini Kit	Susceptible
SZ_2023	100	Hungary	Pest	Budapest	4	47.498333	19.040833	2023	2023.07.14	BG-Sentinel	*Culex pipiens*	Qiagen - QIAamp Viral RNA Mini Kit	Heterozygous
SZ_2023	101	Hungary	Pest	Budapest	4	47.498333	19.040833	2023	2023.07.14	BG-Sentinel	*Culex pipiens*	Qiagen - QIAamp Viral RNA Mini Kit	Heterozygous
SZ_2023	102	Hungary	Pest	Budapest	4	47.498333	19.040833	2023	2023.07.14	BG-Sentinel	*Culex pipiens*	Qiagen - QIAamp Viral RNA Mini Kit	Susceptible
SZ_2022	93	Hungary	Pest	Vác	5	47.775278	19.131111	2022	2022.06.19	BG-Sentinel	*Culex pipiens*	Qiagen - QIAamp Viral RNA Mini Kit	Heterozygous
SZ_2022	105	Hungary	Pest	Vác	5	47.775278	19.131111	2022	2022.06.26	BG-Sentinel	*Culex pipiens*	Qiagen - QIAamp Viral RNA Mini Kit	Heterozygous
SZ_2022	666	Hungary	Pest	Vác	5	47.775278	19.131111	2022	2022.07.24	BG-Sentinel	*Culex pipiens*	Qiagen - QIAamp Viral RNA Mini Kit	Susceptible
SZ_2022	698	Hungary	Pest	Vác	5	47.775278	19.131111	2022	2022.07.31	BG-Sentinel	*Culex pipiens*	Qiagen - QIAamp Viral RNA Mini Kit	Resistant
SZ_2022	706	Hungary	Pest	Vác	5	47.775278	19.131111	2022	2022.08.07	BG-Sentinel	*Culex pipiens*	Qiagen - QIAamp Viral RNA Mini Kit	Resistant
SZ_2022	733	Hungary	Pest	Vác	5	47.775278	19.131111	2022	2022.08.14	BG-Sentinel	*Culex pipiens*	Qiagen - QIAamp Viral RNA Mini Kit	Susceptible
SZ_2022	746	Hungary	Pest	Vác	5	47.775278	19.131111	2022	2022.08.21	BG-Sentinel	*Culex pipiens*	Qiagen - QIAamp Viral RNA Mini Kit	Susceptible
SZ_2022	201	Hungary	Pest	Vác	5	47.775278	19.131111	2022	2022.07.03.	BG-Sentinel	*Culex pipiens*	Qiagen - QIAamp Viral RNA Mini Kit	Susceptible
SZ_2022	218	Hungary	Pest	Vác	5	47.775278	19.131111	2022	2022.07.10.	BG-Sentinel	*Culex pipiens*	Qiagen - QIAamp Viral RNA Mini Kit	Heterozygous
VT_2023	201	Hungary	Pest	Vác	5	47.775278	19.131111	2023	2023.07.02.	BG-Sentinel	*Culex pipiens*	Qiagen - QIAamp Viral RNA Mini Kit	Heterozygous
VT_2023	208	Hungary	Pest	Vác	5	47.775278	19.131111	2023	2023.07.09.	BG-Sentinel	*Culex pipiens*	Qiagen - QIAamp Viral RNA Mini Kit	Resistant
BGC	239	Hungary	Somogy	Barcs	8	45.958492	17.468648	2021	2021.08.08	BG-Sentinel	*Culex pipiens*	Zymo - Quick-DNA Miniprep Plus Kit	Heterozygous
BGC	319	Hungary	Somogy	Barcs	8	45.958492	17.468648	2021	2021.07.20.	BG-Sentinel	*Culex pipiens*	Zymo - Quick-DNA Miniprep Plus Kit	Susceptible
BGC	300	Hungary	Somogy	Barcs	8	45.958492	17.468648	2021	2021.07.21.	BG-Sentinel	*Culex pipiens*	Zymo - Quick-DNA Miniprep Plus Kit	Heterozygous
BGC	302	Hungary	Somogy	Barcs	8	45.958492	17.468648	2021	2021.07.21.	BG-Sentinel	*Culex pipiens*	Zymo - Quick-DNA Miniprep Plus Kit	Heterozygous
BGC	305	Hungary	Somogy	Barcs	8	45.958492	17.468648	2021	2021.07.21.	BG-Sentinel	*Culex pipiens*	Zymo - Quick-DNA Miniprep Plus Kit	Heterozygous
BGC	301	Hungary	Somogy	Barcs	8	45.958492	17.468648	2021	2021.07.21.	BG-Sentinel	*Culex pipiens*	Zymo - Quick-DNA Miniprep Plus Kit	Susceptible
BGC	306	Hungary	Somogy	Barcs	8	45.958492	17.468648	2021	2021.07.21.	BG-Sentinel	*Culex pipiens*	Zymo - Quick-DNA Miniprep Plus Kit	Susceptible
BGC	289	Hungary	Somogy	Barcs	8	45.958492	17.468648	2021	2021.07.22.	BG-Sentinel	*Culex pipiens*	Zymo - Quick-DNA Miniprep Plus Kit	Susceptible
BGC	284	Hungary	Somogy	Barcs	8	45.958492	17.468648	2021	2021.07.23.	BG-Sentinel	*Culex pipiens*	Zymo - Quick-DNA Miniprep Plus Kit	Susceptible
BGC	211	Hungary	Somogy	Barcs	8	45.958492	17.468648	2021	2021.07.24.	BG-Sentinel	*Culex pipiens*	Zymo - Quick-DNA Miniprep Plus Kit	Susceptible
BGC	212	Hungary	Somogy	Barcs	8	45.958492	17.468648	2021	2021.07.24.	BG-Sentinel	*Culex pipiens*	Zymo - Quick-DNA Miniprep Plus Kit	Susceptible
BGC	189	Hungary	Somogy	Barcs	8	45.958492	17.468648	2021	2021.07.25.	BG-Sentinel	*Culex pipiens*	Zymo - Quick-DNA Miniprep Plus Kit	Susceptible

BGC	190	Hungary	Somogy	Barcs	8	45.958492	17.468648	2021	2021.07.25.	BG-Sentinel	*Culex pipiens*	Zymo - Quick-DNA Miniprep Plus Kit	Susceptible
BGC	142	Hungary	Somogy	Barcs	8	45.958492	17.468648	2021	2021.08.04.	BG-Sentinel	*Culex pipiens*	Zymo - Quick-DNA Miniprep Plus Kit	Heterozygous
BGC	203	Hungary	Somogy	Barcs	8	45.958492	17.468648	2021	2021.08.04.	BG-Sentinel	*Culex pipiens*	Zymo - Quick-DNA Miniprep Plus Kit	Susceptible
BGC	141	Hungary	Somogy	Barcs	8	45.958492	17.468648	2021	2021.08.04.	BG-Sentinel	*Culex pipiens*	Zymo - Quick-DNA Miniprep Plus Kit	Susceptible
BGC	217	Hungary	Somogy	Barcs	8	45.958492	17.468648	2021	2021.08.06.	BG-Sentinel	*Culex pipiens*	Zymo - Quick-DNA Miniprep Plus Kit	Heterozygous
BGC	227	Hungary	Somogy	Barcs	8	45.958492	17.468648	2021	2021.08.07.	BG-Sentinel	*Culex pipiens*	Zymo - Quick-DNA Miniprep Plus Kit	Heterozygous
BGC	238	Hungary	Somogy	Barcs	8	45.958492	17.468648	2021	2021.08.08.	BG-Sentinel	*Culex pipiens*	Zymo - Quick-DNA Miniprep Plus Kit	Susceptible
BGC	177	Hungary	Somogy	Barcs	8	45.958492	17.468648	2021	2021.08.09.	BG-Sentinel	*Culex pipiens*	Zymo - Quick-DNA Miniprep Plus Kit	Resistant
BGC	209	Hungary	Somogy	Barcs	8	45.958492	17.468648	2021	2021.08.10.	BG-Sentinel	*Culex pipiens*	Zymo - Quick-DNA Miniprep Plus Kit	Susceptible
BGC	307	Hungary	Somogy	Barcs	8	45.958492	17.468648	2021	2021.08.11.	BG-Sentinel	*Culex pipiens*	Zymo - Quick-DNA Miniprep Plus Kit	Susceptible
BGC	314	Hungary	Somogy	Barcs	8	45.958492	17.468648	2021	2021.08.12.	BG-Sentinel	*Culex pipiens*	Zymo - Quick-DNA Miniprep Plus Kit	Susceptible
BGC	260	Hungary	Somogy	Barcs	8	45.958492	17.468648	2021	2021.08.13.	BG-Sentinel	*Culex pipiens*	Zymo - Quick-DNA Miniprep Plus Kit	Susceptible
BGC	242	Hungary	Somogy	Barcs	8	45.958492	17.468648	2021	2021.08.15.	BG-Sentinel	*Culex pipiens*	Zymo - Quick-DNA Miniprep Plus Kit	Heterozygous
BGC	257	Hungary	Somogy	Barcs	8	45.958492	17.468648	2021	2021.08.16.	BG-Sentinel	*Culex pipiens*	Zymo - Quick-DNA Miniprep Plus Kit	Susceptible
BGC	275	Hungary	Somogy	Barcs	8	45.958492	17.468648	2021	2021.08.17.	BG-Sentinel	*Culex pipiens*	Zymo - Quick-DNA Miniprep Plus Kit	Heterozygous
BGC	279	Hungary	Somogy	Barcs	8	45.958492	17.468648	2021	2021.08.18.	BG-Sentinel	*Culex pipiens*	Zymo - Quick-DNA Miniprep Plus Kit	Susceptible
BGC	267	Hungary	Somogy	Barcs	8	45.958492	17.468648	2021	2021.08.19.	BG-Sentinel	*Culex pipiens*	Zymo - Quick-DNA Miniprep Plus Kit	Heterozygous
BGC	197	Hungary	Somogy	Barcs	8	45.958492	17.468648	2021	2021.08.24.	BG-Sentinel	*Culex pipiens*	Zymo - Quick-DNA Miniprep Plus Kit	Susceptible
BGC	272	Hungary	Somogy	Barcs	8	45.958492	17.468648	2021	2021.08.25.	BG-Sentinel	*Culex pipiens*	Zymo - Quick-DNA Miniprep Plus Kit	Heterozygous
BGC	175	Hungary	Somogy	Barcs	8	45.958492	17.468648	2021	2021.08.28.	BG-Sentinel	*Culex pipiens*	Zymo - Quick-DNA Miniprep Plus Kit	Heterozygous
BGC	161	Hungary	Somogy	Barcs	8	45.958492	17.468648	2021	2021.09.01.	BG-Sentinel	*Culex pipiens*	Zymo - Quick-DNA Miniprep Plus Kit	Susceptible
BGC	166	Hungary	Somogy	Barcs	8	45.958492	17.468648	2021	2021.09.03.	BG-Sentinel	*Culex pipiens*	Zymo - Quick-DNA Miniprep Plus Kit	Heterozygous
BGC	132	Hungary	Somogy	Barcs	8	45.958492	17.468648	2021	2021.09.05.	BG-Sentinel	*Culex pipiens*	Zymo - Quick-DNA Miniprep Plus Kit	Susceptible
BGC	128	Hungary	Somogy	Barcs	8	45.958492	17.468648	2021	2021.09.15.	BG-Sentinel	*Culex pipiens*	Zymo - Quick-DNA Miniprep Plus Kit	Susceptible
BGC	157	Hungary	Somogy	Barcs	8	45.958492	17.468648	2021	2021.09.16.	BG-Sentinel	*Culex pipiens*	Zymo - Quick-DNA Miniprep Plus Kit	Heterozygous
BGC	154	Hungary	Somogy	Barcs	8	45.958492	17.468648	2021	2021.09.28.	BG-Sentinel	*Culex pipiens*	Zymo - Quick-DNA Miniprep Plus Kit	Susceptible
BGC	325	Hungary	Somogy	Barcs	8	45.958492	17.468648	2021	2021.10.07.	BG-Sentinel	*Culex pipiens*	Zymo - Quick-DNA Miniprep Plus Kit	Susceptible
BGC	330	Hungary	Somogy	Barcs	8	45.958492	17.468648	2021	2021.10.15.	BG-Sentinel	*Culex pipiens*	Zymo - Quick-DNA Miniprep Plus Kit	Susceptible

BGC	333	Hungary	Somogy	Barcs	8	45.958492	17.468648	2021	2021.10.18.	BG-Sentinel	*Culex pipiens*	Zymo - Quick-DNA Miniprep Plus Kit	Susceptible
BGC	334	Hungary	Somogy	Barcs	8	45.958492	17.468648	2021	2021.10.29.	BG-Sentinel	*Culex pipiens*	Zymo - Quick-DNA Miniprep Plus Kit	Susceptible
BGC	339	Hungary	Somogy	Barcs	8	45.958492	17.468648	2022	2022.06.21.	BG-Sentinel	*Culex pipiens*	Zymo - Quick-DNA Miniprep Plus Kit	Susceptible
BGC	342	Hungary	Somogy	Barcs	8	45.958492	17.468648	2022	2022.06.22.	BG-Sentinel	*Culex pipiens*	Zymo - Quick-DNA Miniprep Plus Kit	Susceptible
BGC	344	Hungary	Somogy	Barcs	8	45.958492	17.468648	2022	2022.06.23.	BG-Sentinel	*Culex pipiens*	Zymo - Quick-DNA Miniprep Plus Kit	Susceptible
BGC	367	Hungary	Somogy	Barcs	8	45.958492	17.468648	2022	2022.06.25.	BG-Sentinel	*Culex pipiens*	Zymo - Quick-DNA Miniprep Plus Kit	Susceptible
BGC	359	Hungary	Somogy	Barcs	8	45.958492	17.468648	2022	2022.07.04.	BG-Sentinel	*Culex pipiens*	Zymo - Quick-DNA Miniprep Plus Kit	Heterozygous
AIM	264	Hungary	Baranya	Pécs	7	46.070833	18.233056	2022	2022.08.26.	EVS	*Aedes albopictus*	Zymo - Quick-DNA Miniprep Plus Kit	Susceptible/Susceptible
AIM	283	Hungary	Baranya	Pécs	7	46.070833	18.233056	2022	2022.09.01.	EVS	*Aedes albopictus*	Zymo - Quick-DNA Miniprep Plus Kit	Susceptible/Susceptible
AIM	300	Hungary	Baranya	Pécs	7	46.070833	18.233056	2022	2022.09.15.	EVS	*Aedes albopictus*	Zymo - Quick-DNA Miniprep Plus Kit	Susceptible/Susceptible
AIM	301	Hungary	Baranya	Pécs	7	46.070833	18.233056	2022	2022.09.15.	EVS	*Aedes albopictus*	Zymo - Quick-DNA Miniprep Plus Kit	Susceptible/Susceptible
AIM	622	Hungary	Baranya	Pécs	7	46.070833	18.233056	2023	2023.10.12	EVS	*Aedes albopictus*	Zymo - Quick-DNA Miniprep Plus Kit	Susceptible/Susceptible
AIM	623	Hungary	Baranya	Pécs	7	46.070833	18.233056	2023	2023.10.12	EVS	*Aedes albopictus*	Zymo - Quick-DNA Miniprep Plus Kit	Susceptible/Susceptible
AIM	624	Hungary	Baranya	Pécs	7	46.070833	18.233056	2023	2023.10.12	EVS	*Aedes albopictus*	Zymo - Quick-DNA Miniprep Plus Kit	Susceptible/Susceptible
AIM	625	Hungary	Baranya	Pécs	7	46.070833	18.233056	2023	2023.10.12	EVS	*Aedes albopictus*	Zymo - Quick-DNA Miniprep Plus Kit	Susceptible/Susceptible
AIM	626	Hungary	Baranya	Pécs	7	46.070833	18.233056	2023	2023.10.12	EVS	*Aedes albopictus*	Zymo - Quick-DNA Miniprep Plus Kit	Susceptible/Susceptible
AIM	627	Hungary	Baranya	Pécs	7	46.070833	18.233056	2023	2023.10.12	EVS	*Aedes albopictus*	Zymo - Quick-DNA Miniprep Plus Kit	Susceptible/Susceptible
AIM	628	Hungary	Baranya	Pécs	7	46.070833	18.233056	2023	2023.10.12	EVS	*Aedes albopictus*	Zymo - Quick-DNA Miniprep Plus Kit	Susceptible/Susceptible
AIM	629	Hungary	Baranya	Pécs	7	46.070833	18.233056	2023	2023.10.12	EVS	*Aedes albopictus*	Zymo - Quick-DNA Miniprep Plus Kit	Susceptible/Susceptible
AIM	630	Hungary	Baranya	Pécs	7	46.070833	18.233056	2023	2023.10.12	EVS	*Aedes albopictus*	Zymo - Quick-DNA Miniprep Plus Kit	Susceptible/Susceptible
AIM	521	Hungary	Baranya	Pécs	7	46.070833	18.233056	2023	2023.08.02.	EVS	*Aedes albopictus*	Zymo - Quick-DNA Miniprep Plus Kit	Susceptible/Susceptible
AIM	524	Hungary	Baranya	Pécs	7	46.070833	18.233056	2023	2023.08.02.	EVS	*Aedes albopictus*	Zymo - Quick-DNA Miniprep Plus Kit	Susceptible/Susceptible
AIM	533	Hungary	Baranya	Pécs	7	46.070833	18.233056	2023	2023.08.30.	EVS	*Aedes albopictus*	Zymo - Quick-DNA Miniprep Plus Kit	Susceptible/Susceptible
AIM	534	Hungary	Baranya	Pécs	7	46.070833	18.233056	2023	2023.08.30.	EVS	*Aedes albopictus*	Zymo - Quick-DNA Miniprep Plus Kit	Susceptible/Susceptible
AIM	535	Hungary	Baranya	Pécs	7	46.070833	18.233056	2023	2023.08.30.	EVS	*Aedes albopictus*	Zymo - Quick-DNA Miniprep Plus Kit	Susceptible/Susceptible
AIM	540	Hungary	Baranya	Pécs	7	46.070833	18.233056	2023	2023.09.06.	EVS	*Aedes albopictus*	Zymo - Quick-DNA Miniprep Plus Kit	Susceptible/Susceptible
AIM	541	Hungary	Baranya	Pécs	7	46.070833	18.233056	2023	2023.09.06.	EVS	*Aedes albopictus*	Zymo - Quick-DNA Miniprep Plus Kit	Susceptible/Susceptible
AIM	544	Hungary	Baranya	Pécs	7	46.070833	18.233056	2023	2023.09.06.	EVS	*Aedes albopictus*	Zymo - Quick-DNA Miniprep Plus Kit	Susceptible/Susceptible

AIM	545	Hungary	Baranya	Pécs	7	46.070833	18.233056	2023	2023.09.06.	EVS	*Aedes albopictus*	Zymo - Quick-DNA Miniprep Plus Kit	Susceptible/Susceptible
AIM	554	Hungary	Baranya	Pécs	7	46.070833	18.233056	2023	2023.09.14.	EVS	*Aedes albopictus*	Zymo - Quick-DNA Miniprep Plus Kit	Susceptible/Susceptible
AIM	555	Hungary	Baranya	Pécs	7	46.070833	18.233056	2023	2023.09.14.	EVS	*Aedes albopictus*	Zymo - Quick-DNA Miniprep Plus Kit	Susceptible/Susceptible
AIM	559	Hungary	Baranya	Pécs	7	46.070833	18.233056	2023	2023.09.14.	EVS	*Aedes albopictus*	Zymo - Quick-DNA Miniprep Plus Kit	Susceptible/Susceptible
AIM	560	Hungary	Baranya	Pécs	7	46.070833	18.233056	2023	2023.09.14.	EVS	*Aedes albopictus*	Zymo - Quick-DNA Miniprep Plus Kit	Susceptible/Susceptible
AIM	561	Hungary	Baranya	Pécs	7	46.070833	18.233056	2023	2023.09.14.	EVS	*Aedes albopictus*	Zymo - Quick-DNA Miniprep Plus Kit	Susceptible/Susceptible
AIM	562	Hungary	Baranya	Pécs	7	46.070833	18.233056	2023	2023.09.14.	EVS	*Aedes albopictus*	Zymo - Quick-DNA Miniprep Plus Kit	Susceptible/Susceptible
AIM	563	Hungary	Baranya	Pécs	7	46.070833	18.233056	2023	2023.09.14.	EVS	*Aedes albopictus*	Zymo - Quick-DNA Miniprep Plus Kit	Susceptible/Susceptible
AIM	564	Hungary	Baranya	Pécs	7	46.070833	18.233056	2023	2023.09.21.	EVS	*Aedes albopictus*	Zymo - Quick-DNA Miniprep Plus Kit	Susceptible/Susceptible
AIM	565	Hungary	Baranya	Pécs	7	46.070833	18.233056	2023	2023.09.21.	EVS	*Aedes albopictus*	Zymo - Quick-DNA Miniprep Plus Kit	Susceptible/Susceptible
AIM	566	Hungary	Baranya	Pécs	7	46.070833	18.233056	2023	2023.09.21.	EVS	*Aedes albopictus*	Zymo - Quick-DNA Miniprep Plus Kit	Susceptible/Susceptible
AIM	571	Hungary	Baranya	Pécs	7	46.070833	18.233056	2023	2023.09.21.	EVS	*Aedes albopictus*	Zymo - Quick-DNA Miniprep Plus Kit	Susceptible/Susceptible
AIM	573	Hungary	Baranya	Pécs	7	46.070833	18.233056	2023	2023.09.21.	EVS	*Aedes albopictus*	Zymo - Quick-DNA Miniprep Plus Kit	Susceptible/Susceptible
AIM	582	Hungary	Baranya	Pécs	7	46.070833	18.233056	2023	2023.09.28.	EVS	*Aedes albopictus*	Zymo - Quick-DNA Miniprep Plus Kit	Susceptible/Susceptible
AIM	583	Hungary	Baranya	Pécs	7	46.070833	18.233056	2023	2023.09.28.	EVS	*Aedes albopictus*	Zymo - Quick-DNA Miniprep Plus Kit	Susceptible/Susceptible
AIM	585	Hungary	Baranya	Pécs	7	46.070833	18.233056	2023	2023.09.28.	EVS	*Aedes albopictus*	Zymo - Quick-DNA Miniprep Plus Kit	Susceptible/Susceptible
AIM	586	Hungary	Baranya	Pécs	7	46.070833	18.233056	2023	2023.09.28.	EVS	*Aedes albopictus*	Zymo - Quick-DNA Miniprep Plus Kit	Susceptible/Susceptible
AIM	587	Hungary	Baranya	Pécs	7	46.070833	18.233056	2023	2023.09.28.	EVS	*Aedes albopictus*	Zymo - Quick-DNA Miniprep Plus Kit	Susceptible/Susceptible
AIM	588	Hungary	Baranya	Pécs	7	46.070833	18.233056	2023	2023.09.28.	EVS	*Aedes albopictus*	Zymo - Quick-DNA Miniprep Plus Kit	Susceptible/Susceptible
AIM	589	Hungary	Baranya	Pécs	7	46.070833	18.233056	2023	2023.09.28.	EVS	*Aedes albopictus*	Zymo - Quick-DNA Miniprep Plus Kit	Susceptible/Susceptible
AIM	590	Hungary	Baranya	Pécs	7	46.070833	18.233056	2023	2023.09.28.	EVS	*Aedes albopictus*	Zymo - Quick-DNA Miniprep Plus Kit	Susceptible/Susceptible
AIM	591	Hungary	Baranya	Pécs	7	46.070833	18.233056	2023	2023.09.28.	EVS	*Aedes albopictus*	Zymo - Quick-DNA Miniprep Plus Kit	Susceptible/Susceptible
AIM	592	Hungary	Baranya	Pécs	7	46.070833	18.233056	2023	2023.09.28.	EVS	*Aedes albopictus*	Zymo - Quick-DNA Miniprep Plus Kit	Susceptible/Susceptible
AIM	593	Hungary	Baranya	Pécs	7	46.070833	18.233056	2023	2023.09.28.	EVS	*Aedes albopictus*	Zymo - Quick-DNA Miniprep Plus Kit	Susceptible/Susceptible
AIM	606	Hungary	Baranya	Pécs	7	46.070833	18.233056	2023	2023.10.05.	EVS	*Aedes albopictus*	Zymo - Quick-DNA Miniprep Plus Kit	Susceptible/Susceptible
AIM	607	Hungary	Baranya	Pécs	7	46.070833	18.233056	2023	2023.10.05.	EVS	*Aedes albopictus*	Zymo - Quick-DNA Miniprep Plus Kit	Susceptible/Susceptible
AIM	611	Hungary	Baranya	Pécs	7	46.070833	18.233056	2023	2023.10.05.	EVS	*Aedes albopictus*	Zymo - Quick-DNA Miniprep Plus Kit	Susceptible/Susceptible
AIM	612	Hungary	Baranya	Pécs	7	46.070833	18.233056	2023	2023.10.05.	EVS	*Aedes albopictus*	Zymo - Quick-DNA Miniprep Plus Kit	Susceptible/Susceptible

AIM	613	Hungary	Baranya	Pécs	7	46.070833	18.233056	2023	2023.10.05.	EVS	*Aedes albopictus*	Zymo - Quick-DNA Miniprep Plus Kit	Susceptible/Susceptible
SZ_2022	57	Hungary	Pest	Budapest	4	47.498333	19.040833	2022	2022.05.16.	BG-Sentinel	*Aedes albopictus*	Qiagen - QIAamp Viral RNA Mini Kit	Susceptible/Susceptible
SZ_2022	71	Hungary	Pest	Budapest	4	47.498333	19.040833	2022	2022.05.30.	BG-Sentinel	*Aedes albopictus*	Qiagen - QIAamp Viral RNA Mini Kit	Susceptible/Susceptible
SZ_2022	20	Hungary	Pest	Budapest	4	47.498333	19.040833	2022	2022.06.09.	BG-Sentinel	*Aedes albopictus*	Qiagen - QIAamp Viral RNA Mini Kit	Susceptible/Susceptible
SZ_2022	21	Hungary	Pest	Budapest	4	47.498333	19.040833	2022	2022.06.09.	BG-Sentinel	*Aedes albopictus*	Qiagen - QIAamp Viral RNA Mini Kit	Susceptible/Susceptible
SZ_2022	22	Hungary	Pest	Budapest	4	47.498333	19.040833	2022	2022.06.09.	BG-Sentinel	*Aedes albopictus*	Qiagen - QIAamp Viral RNA Mini Kit	Susceptible/Susceptible
SZ_2022	23	Hungary	Pest	Budapest	4	47.498333	19.040833	2022	2022.06.09.	BG-Sentinel	*Aedes albopictus*	Qiagen - QIAamp Viral RNA Mini Kit	Susceptible/Susceptible
SZ_2022	24	Hungary	Pest	Budapest	4	47.498333	19.040833	2022	2022.06.09.	BG-Sentinel	*Aedes albopictus*	Qiagen - QIAamp Viral RNA Mini Kit	Susceptible/Susceptible
SZ_2022	25	Hungary	Pest	Budapest	4	47.498333	19.040833	2022	2022.06.09.	BG-Sentinel	*Aedes albopictus*	Qiagen - QIAamp Viral RNA Mini Kit	Susceptible/Susceptible
SZ_2022	26	Hungary	Pest	Budapest	4	47.498333	19.040833	2022	2022.06.09.	BG-Sentinel	*Aedes albopictus*	Qiagen - QIAamp Viral RNA Mini Kit	Susceptible/Susceptible
SZ_2022	27	Hungary	Pest	Budapest	4	47.498333	19.040833	2022	2022.06.09.	BG-Sentinel	*Aedes albopictus*	Qiagen - QIAamp Viral RNA Mini Kit	Susceptible/Susceptible
SZ_2022	28	Hungary	Pest	Budapest	4	47.498333	19.040833	2022	2022.06.09.	BG-Sentinel	*Aedes albopictus*	Qiagen - QIAamp Viral RNA Mini Kit	Susceptible/Susceptible
SZ_2022	29	Hungary	Pest	Budapest	4	47.498333	19.040833	2022	2022.06.09.	BG-Sentinel	*Aedes albopictus*	Qiagen - QIAamp Viral RNA Mini Kit	Susceptible/Susceptible
SZ_2022	150	Hungary	Pest	Budapest	4	47.498333	19.040833	2022	2022.06.27.	BG-Sentinel	*Aedes albopictus*	Qiagen - QIAamp Viral RNA Mini Kit	Susceptible/Susceptible
SZ_2022	856	Hungary	Pest	Budapest	4	47.498333	19.040833	2022	2022.08.01.	BG-Sentinel	*Aedes albopictus*	Qiagen - QIAamp Viral RNA Mini Kit	Susceptible/Susceptible
SZ_2022	857	Hungary	Pest	Budapest	4	47.498333	19.040833	2022	2022.08.01.	BG-Sentinel	*Aedes albopictus*	Qiagen - QIAamp Viral RNA Mini Kit	Susceptible/Susceptible
SZ_2022	863	Hungary	Pest	Budapest	4	47.498333	19.040833	2022	2022.08.08.	BG-Sentinel	*Aedes albopictus*	Qiagen - QIAamp Viral RNA Mini Kit	Susceptible/Susceptible
SZ_2022	872	Hungary	Pest	Budapest	4	47.498333	19.040833	2022	2022.08.15.	BG-Sentinel	*Aedes albopictus*	Qiagen - QIAamp Viral RNA Mini Kit	Susceptible/Susceptible
SZ_2022	909	Hungary	Pest	Budapest	4	47.498333	19.040833	2022	2022.08.29.	BG-Sentinel	*Aedes albopictus*	Qiagen - QIAamp Viral RNA Mini Kit	Susceptible/Susceptible
SZ_2022	1163	Hungary	Pest	Budapest	4	47.498333	19.040833	2022	2022.09.19.	BG-Sentinel	*Aedes albopictus*	Qiagen - QIAamp Viral RNA Mini Kit	Susceptible/Susceptible
SZ_2022	1168	Hungary	Pest	Budapest	4	47.498333	19.040833	2022	2022.09.19.	BG-Sentinel	*Aedes albopictus*	Qiagen - QIAamp Viral RNA Mini Kit	Susceptible/Susceptible
BGC	320	Hungary	Somogy	Barcs	8	45.958492	17.468648	2021	2021.07.20.	BG-Sentinel	*Aedes albopictus*	Zymo - Quick-DNA Miniprep Plus Kit	Susceptible/Susceptible
BGC	299	Hungary	Somogy	Barcs	8	45.958492	17.468648	2021	2021.07.21.	BG-Sentinel	*Aedes albopictus*	Zymo - Quick-DNA Miniprep Plus Kit	Susceptible/Susceptible
BGC	285	Hungary	Somogy	Barcs	8	45.958492	17.468648	2021	2021.07.23.	BG-Sentinel	*Aedes albopictus*	Zymo - Quick-DNA Miniprep Plus Kit	Susceptible/Susceptible
BGC	213	Hungary	Somogy	Barcs	8	45.958492	17.468648	2021	2021.07.24.	BG-Sentinel	*Aedes albopictus*	Zymo - Quick-DNA Miniprep Plus Kit	Susceptible/Susceptible
BGC	102	Hungary	Somogy	Barcs	8	45.958492	17.468648	2021	2021.08.02.	BG-Sentinel	*Aedes albopictus*	Zymo - Quick-DNA Miniprep Plus Kit	Susceptible/Susceptible
BGC	138	Hungary	Somogy	Barcs	8	45.958492	17.468648	2021	2021.08.04.	BG-Sentinel	*Aedes albopictus*	Zymo - Quick-DNA Miniprep Plus Kit	Susceptible/Susceptible
BGC	201	Hungary	Somogy	Barcs	8	45.958492	17.468648	2021	2021.08.04.	BG-Sentinel	*Aedes albopictus*	Zymo - Quick-DNA Miniprep Plus Kit	Susceptible/Susceptible

BGC	220	Hungary	Somogy	Barcs	8	45.958492	17.468648	2021	2021.08.06.	BG-Sentinel	*Aedes albopictus*	Zymo - Quick-DNA Miniprep Plus Kit	Susceptible/Susceptible
BGC	176	Hungary	Somogy	Barcs	8	45.958492	17.468648	2021	2021.08.09.	BG-Sentinel	*Aedes albopictus*	Zymo - Quick-DNA Miniprep Plus Kit	Susceptible/Susceptible
BGC	206	Hungary	Somogy	Barcs	8	45.958492	17.468648	2021	2021.08.10.	BG-Sentinel	*Aedes albopictus*	Zymo - Quick-DNA Miniprep Plus Kit	Susceptible/Susceptible
BGC	309	Hungary	Somogy	Barcs	8	45.958492	17.468648	2021	2021.08.11.	BG-Sentinel	*Aedes albopictus*	Zymo - Quick-DNA Miniprep Plus Kit	Susceptible/Susceptible
BGC	263	Hungary	Somogy	Barcs	8	45.958492	17.468648	2021	2021.08.13.	BG-Sentinel	*Aedes albopictus*	Zymo - Quick-DNA Miniprep Plus Kit	Susceptible/Susceptible
BGC	243	Hungary	Somogy	Barcs	8	45.958492	17.468648	2021	2021.08.15.	BG-Sentinel	*Aedes albopictus*	Zymo - Quick-DNA Miniprep Plus Kit	Susceptible/Susceptible
BGC	281	Hungary	Somogy	Barcs	8	45.958492	17.468648	2021	2021.08.18.	BG-Sentinel	*Aedes albopictus*	Zymo - Quick-DNA Miniprep Plus Kit	Susceptible/Susceptible
BGC	264	Hungary	Somogy	Barcs	8	45.958492	17.468648	2021	2021.08.19.	BG-Sentinel	*Aedes albopictus*	Zymo - Quick-DNA Miniprep Plus Kit	Susceptible/Susceptible
BGC	292	Hungary	Somogy	Barcs	8	45.958492	17.468648	2021	2021.08.21.	BG-Sentinel	*Aedes albopictus*	Zymo - Quick-DNA Miniprep Plus Kit	Susceptible/Susceptible
BGC	199	Hungary	Somogy	Barcs	8	45.958492	17.468648	2021	2021.08.24.	BG-Sentinel	*Aedes albopictus*	Zymo - Quick-DNA Miniprep Plus Kit	Susceptible/Susceptible
BGC	271	Hungary	Somogy	Barcs	8	45.958492	17.468648	2021	2021.08.25.	BG-Sentinel	*Aedes albopictus*	Zymo - Quick-DNA Miniprep Plus Kit	Susceptible/Susceptible
BGC	170	Hungary	Somogy	Barcs	8	45.958492	17.468648	2021	2021.08.26.	BG-Sentinel	*Aedes albopictus*	Zymo - Quick-DNA Miniprep Plus Kit	Susceptible/Susceptible
BGC	160	Hungary	Somogy	Barcs	8	45.958492	17.468648	2021	2021.09.01.	BG-Sentinel	*Aedes albopictus*	Zymo - Quick-DNA Miniprep Plus Kit	Susceptible/Susceptible
BGC	150	Hungary	Somogy	Barcs	8	45.958492	17.468648	2021	2021.09.02.	BG-Sentinel	*Aedes albopictus*	Zymo - Quick-DNA Miniprep Plus Kit	Susceptible/Susceptible
BGC	130	Hungary	Somogy	Barcs	8	45.958492	17.468648	2021	2021.09.05.	BG-Sentinel	*Aedes albopictus*	Zymo - Quick-DNA Miniprep Plus Kit	Susceptible/Susceptible
BGC	146	Hungary	Somogy	Barcs	8	45.958492	17.468648	2021	2021.09.10.	BG-Sentinel	*Aedes albopictus*	Zymo - Quick-DNA Miniprep Plus Kit	Susceptible/Susceptible
BGC	155	Hungary	Somogy	Barcs	8	45.958492	17.468648	2021	2021.09.16.	BG-Sentinel	*Aedes albopictus*	Zymo - Quick-DNA Miniprep Plus Kit	Susceptible/Susceptible
BGC	111	Hungary	Somogy	Barcs	8	45.958492	17.468648	2021	2021.09.20.	BG-Sentinel	*Aedes albopictus*	Zymo - Quick-DNA Miniprep Plus Kit	Susceptible/Susceptible
BGC	124	Hungary	Somogy	Barcs	8	45.958492	17.468648	2021	2021.09.23.	BG-Sentinel	*Aedes albopictus*	Zymo - Quick-DNA Miniprep Plus Kit	Susceptible/Susceptible
BGC	134	Hungary	Somogy	Barcs	8	45.958492	17.468648	2021	2021.09.25.	BG-Sentinel	*Aedes albopictus*	Zymo - Quick-DNA Miniprep Plus Kit	Susceptible/Susceptible
BGC	152	Hungary	Somogy	Barcs	8	45.958492	17.468648	2021	2021.09.28.	BG-Sentinel	*Aedes albopictus*	Zymo - Quick-DNA Miniprep Plus Kit	Susceptible/Susceptible
BGC	147	Hungary	Somogy	Barcs	8	45.958492	17.468648	2021	2021.10.04.	BG-Sentinel	*Aedes albopictus*	Zymo - Quick-DNA Miniprep Plus Kit	Susceptible/Susceptible
BGC	328	Hungary	Somogy	Barcs	8	45.958492	17.468648	2021	2021.10.15.	BG-Sentinel	*Aedes albopictus*	Zymo - Quick-DNA Miniprep Plus Kit	Susceptible/Susceptible
BGC	337	Hungary	Somogy	Barcs	8	45.958492	17.468648	2021	2021.10.21.	BG-Sentinel	*Aedes albopictus*	Zymo - Quick-DNA Miniprep Plus Kit	Susceptible/Susceptible
BGC	345	Hungary	Somogy	Barcs	8	45.958492	17.468648	2022	2022.06.23.	BG-Sentinel	*Aedes albopictus*	Zymo - Quick-DNA Miniprep Plus Kit	Susceptible/Susceptible
BGC	372	Hungary	Somogy	Barcs	8	45.958492	17.468648	2022	2022.06.29.	BG-Sentinel	*Aedes albopictus*	Zymo - Quick-DNA Miniprep Plus Kit	Susceptible/Susceptible
BGC	357	Hungary	Somogy	Barcs	8	45.958492	17.468648	2022	2022.07.08.	BG-Sentinel	*Aedes albopictus*	Zymo - Quick-DNA Miniprep Plus Kit	Susceptible/Susceptible
BGC	350	Hungary	Somogy	Barcs	8	45.958492	17.468648	2022	2022.07.09.	BG-Sentinel	*Aedes albopictus*	Zymo - Quick-DNA Miniprep Plus Kit	Susceptible/Susceptible

BGC	492	Hungary	Somogy	Barcs	8	45.958492	17.468648	2022	2022.07.22.	BG-Sentinel	*Aedes albopictus*	Zymo - Quick-DNA Miniprep Plus Kit	Susceptible/Susceptible
BGC	478	Hungary	Somogy	Barcs	8	45.958492	17.468648	2022	2022.07.25.	BG-Sentinel	*Aedes albopictus*	Zymo - Quick-DNA Miniprep Plus Kit	Susceptible/Susceptible
BGC	458	Hungary	Somogy	Barcs	8	45.958492	17.468648	2022	2022.07.29.	BG-Sentinel	*Aedes albopictus*	Zymo - Quick-DNA Miniprep Plus Kit	Susceptible/Susceptible
BGC	453	Hungary	Somogy	Barcs	8	45.958492	17.468648	2022	2022.07.31.	BG-Sentinel	*Aedes albopictus*	Zymo - Quick-DNA Miniprep Plus Kit	Susceptible/Susceptible
BGC	437	Hungary	Somogy	Barcs	8	45.958492	17.468648	2022	2022.08.04.	BG-Sentinel	*Aedes albopictus*	Zymo - Quick-DNA Miniprep Plus Kit	Susceptible/Susceptible
BGC	423	Hungary	Somogy	Barcs	8	45.958492	17.468648	2022	2022.08.08.	BG-Sentinel	*Aedes albopictus*	Zymo - Quick-DNA Miniprep Plus Kit	Susceptible/Susceptible
BGC	414	Hungary	Somogy	Barcs	8	45.958492	17.468648	2022	2022.08.10.	BG-Sentinel	*Aedes albopictus*	Zymo - Quick-DNA Miniprep Plus Kit	Susceptible/Susceptible
BGC	399	Hungary	Somogy	Barcs	8	45.958492	17.468648	2022	2022.08.14.	BG-Sentinel	*Aedes albopictus*	Zymo - Quick-DNA Miniprep Plus Kit	Susceptible/Susceptible
BGC	393	Hungary	Somogy	Barcs	8	45.958492	17.468648	2022	2022.08.17.	BG-Sentinel	*Aedes albopictus*	Zymo - Quick-DNA Miniprep Plus Kit	Susceptible/Susceptible
BGC	527	Hungary	Somogy	Barcs	8	45.958492	17.468648	2022	2022.08.24.	BG-Sentinel	*Aedes albopictus*	Zymo - Quick-DNA Miniprep Plus Kit	Susceptible/Susceptible
BGC	544	Hungary	Somogy	Barcs	8	45.958492	17.468648	2022	2022.09.07.	BG-Sentinel	*Aedes albopictus*	Zymo - Quick-DNA Miniprep Plus Kit	Susceptible/Susceptible
BGC	561	Hungary	Somogy	Barcs	8	45.958492	17.468648	2022	2022.09.13.	BG-Sentinel	*Aedes albopictus*	Zymo - Quick-DNA Miniprep Plus Kit	Susceptible/Susceptible
BGC	589	Hungary	Somogy	Barcs	8	45.958492	17.468648	2022	2022.09.20.	BG-Sentinel	*Aedes albopictus*	Zymo - Quick-DNA Miniprep Plus Kit	Susceptible/Susceptible
BGC	611	Hungary	Somogy	Barcs	8	45.958492	17.468648	2022	2022.09.26.	BG-Sentinel	*Aedes albopictus*	Zymo - Quick-DNA Miniprep Plus Kit	Susceptible/Susceptible
BGC	600	Hungary	Somogy	Barcs	8	45.958492	17.468648	2022	2022.09.28.	BG-Sentinel	*Aedes albopictus*	Zymo - Quick-DNA Miniprep Plus Kit	Susceptible/Susceptible
